# Very rapid cloning, expression and identifying specificity of T-cell receptors for T-cell engineering

**DOI:** 10.1371/journal.pone.0228112

**Published:** 2020-02-10

**Authors:** Shan Zong, Tiejuan Mi, Leo G. Flores, Amir Alpert, Simon Olivares, Krina Patel, Sourindra Maiti, George Mcnamara, Laurence J. N. Cooper, Hiroki Torikai

**Affiliations:** 1 Division of Pediatrics, The University of Texas MD Anderson Cancer Center, Houston, Texas, United States of America; 2 Department of Lymphoma and Myeloma, Division of Cancer Medicine, The University of Texas MD Anderson Cancer Center, Houston, Texas, United States of America; 3 Ziopharm Oncology, Inc., Boston, Massachusetts, United States of America; Universite Paris-Sud, FRANCE

## Abstract

Neoantigens can be predicted and in some cases identified using the data obtained from the whole exome sequencing and transcriptome sequencing of tumor cells. These sequencing data can be coupled with single-cell RNA sequencing for the direct interrogation of the transcriptome, surfaceome, and pairing of αβ T-cell receptors (TCRαβ) from hundreds of single T cells. Using these 2 large datasets, we established a platform for identifying antigens recognized by TCRαβs obtained from single T cells. Our approach is based on the rapid expression of cloned TCRαβ genes as *Sleeping Beauty* transposons and the determination of the introduced TCRαβs’ antigen specificity and avidity using a reporter cell line. The platform enables the very rapid identification of tumor-reactive TCRs for the bioengineering of T cells with redirected specificity.

## Introduction

Two lines of evidence suggest that the immune cell population in the tumor microenvironment is correlated with clinical outcome [1–3]. First, the infiltration of T cells, especially CD8^+^ T cells, is positively correlated with a favorable outcome in many types of cancer [4]. Second, the therapeutic immune checkpoint blockade of CTLA-4 or PDL1/PD1 reinvigorates exhausted tumor-infiltrating lymphocytes (TILs) and has anti-tumor effects in a subset of patients [5]. TILs typically recognize neoantigens that are derived from tumor cell−specific mutations and expressed on tumor cells as peptides in the context of human leukocyte antigens (HLAs) [6]. Recognition of neoantigens by TILs is supported by clinical findings demonstrating that successful immune checkpoint blockade therapy is correlated with high mutation loads in tumor cells [7–10]. That CD8^+^PD1^+^ T cells are enriched in the tumor microenvironment also supports a role for neoantigen-specific TILs as mediators of immune checkpoint blockade [11, 12].

These clinical observations provide a blueprint for using the adoptive transfer of neoantigen-specific T cells with diverse αβ T-cell receptors (TCRαβs) to improve immunotherapy [13]. The use of TILs numerically expanded *ex vivo* has shown promise for the treatment of metastatic melanoma and other solid tumors [14–17]. However, an inherent limitation of TIL-based immunotherapy is that *ex vivo* culture and numeric expansion typically leads to the clonal and/or oligoclonal expansion of terminally differentiated T cells. Together, these clinical data suggest that the administration of “young” T cells that are sourced from peripheral blood and genetically modified to be neoantigen-specific offers an advantage over TIL-based immunotherapy.

The bioengineering of neoantigen-specific T cells requires identifying individual TCRαβs and determining their antigen specificity. Next-generation sequencing (NGS) was used to identify non-synonymous tumor-specific mutations and single-cell RNA sequencing (scRNA-seq) to identify paired full-length TCRαβ sequences [18]. This enabled us to reconstruct tumor-specific TCRs and evaluate their antigen specificity to engineer clinical-grade T cells. This was undertaken by very rapidly constructing a library of TCRαβ genes expressed in DNA plasmids from the *Sleeping Beauty* (SB) transposon/transposase system and then inducing the expression of cloned TCRαβs in a reporter cell line to determine their antigen specificity and avidity. These reporter cells were co-cultured with genetically edited HLA^null^ HEK293 cells and genetically modified with monoallelic HLA and the putative neoantigen as a minigene construct to serve as artificial antigen-presenting cells. This suite of technologies could be used to determine the antigen specificity of TCRs retrieved from primary tumors. In summary, this platform serves as a resource for the very rapid, robust, and high-throughput identification of immunogenic neoantigens and their cognate antigen-specific TCRs.

## Materials and methods

### Ethical statement

Peripheral blood mononuclear cells (PBMCs) were obtained from patients who had provided written informed consent in accordance with a protocol established and approved by MD Anderson’s Institutional Review Board (#LAB07-0296, Acquisition of Peripheral Blood from Healthy Donors). The identities of all patients were kept private. Animals were handled in accordance with strict guidelines established by MD Anderson’s Institutional Animal Care and Use Committee (IACUC), which specifically approved this study (#00001131-RN02, Adoptive Immunotherapy with Genetically Modified T Cell Clones). Mice were housed in pathogen-free conditions and were monitored daily for welfare-related assessments in accordance with IACUC guidelines. Moribund mice were humanely euthanized by CO2 inhalation as per IACUC guidelines. All efforts were made to minimize animal suffering, and inhaled isoflurane was administered for anesthesia as required.

### Cells and cell culture

CD8^+^ T cells were isolated from PBMCs using CD8-specific microbeads (Miltenyi Biotec, Auburn, CA). The Jurkat 76 cell line was kindly provided by Dr. Mirjam Heemskerk (Leiden University, Netherlands). The Jurkat 76 cell line is a subclone of the human CD4^+^ T-acute leukemia cell line, Jurkat, which has disrupted TCRα and TCRβ chains. The Jurkat 76 cell line and its derivatives were maintained in complete medium consisting of RPMI-1640 media (HyClone, GE Healthcare Life Science, Pittsburgh, PA), 10% heat-inactivated fetal bovine serum (FBS; Lonza, Walkersville, MD), and 2 mmol/L L-glutamine (Glutamax-1; Thermo Fisher Scientific, Waltham, MA). The human B-lymphoblastoid cell line 721.221, which lacks HLA-A, -B, and -C expression, and 721.221 cells transduced with HLA-A*02:01 were maintained in complete medium. The HLA-A2^+^ NY-ESO-1−expressing human myeloma cell line U266 was maintained in complete medium. The large T-transformed human embryonic kidney cell line HEK293T was maintained in Iscove’s modified Dulbecco’s medium (Thermo Fisher Scientific) supplemented with 10% FBS and 2 mmol/L L-glutamine. STR profiling was used to verify all cell lines, and all cell lines were periodically tested for Mycoplasma contamination.

### Flow cytometry

Cells were stained with fluorescence-conjugated monoclonal antibodies (mAbs) for 20 minutes at 4°C or with pentamers for 20 minutes at room temperature. Data were acquired using a FACS Caliber (BD Biosciences, San Jose, CA), MACSQuant (Miltenyi Biotec), or iQue (IntelliCyt, Albuquerque, NM) flow cytometer and analyzed using FlowJo version 10.1 (FlowJo, LLC, Ashland, OR) or ForeCyt (IntelliCyt).

Phycoerythrin (PE)- or allophycocyanin (APC)-conjugated CD3 (clone SK7), PE- or APC-conjugated CD8 (clone SK1), APC-conjugated HLA-A, -B, and -C (clone G46-2.6), and PE-conjugated HLA-A2 (clone BB7.2) mAbs were from BD Biosciences. The PE-conjugated CMVpp65 (NLVPMVATV)/HLA-A2 pentamer and PE-conjugated NY-ESO-1 (SLLMWITQV)/HLA-A2 pentamer were from Proimmune (Sarasota, FL).

### Paired CDR3 sequencing of TCR from single T cells

CD8^+^ T cells isolated from PBMCs were stained with the CMV pp65 (NLVPMVATV, HLA-A*0201) pentamer and CD8-specific mAbs. Pentamer^+^CD8^+^ T cells were sorted by fluorescence-activated cell sorting using a FACS Aria (BD Biosciences) into 96-well plates (1 cell per well) preloaded with water and 5 units of proteinase inhibitors (Thermo Fisher Scientific) per well. TCRα and TCRβ CDR3 regions in individual T cells were amplified using a modified protocol described previously [19]. In brief, the sequences of the TCR CDR3 regions were identified through 4 steps (**S3 Fig**):

1) We performed reverse transcription and the first amplification with the One-Step RT-PCR kit (Qiagen, Germantown, MD) using multiplex PCR. We used 38 Vα primers (0.06 μM each) and 1 Cα region primer (0.3 μM) to reverse-transcribe and amplify TCRα CDR3 regions (**S1 Table**). We used 36 Vβ primers (0.06 μM each) and 1 Cβ region primer (0.3 μM) to reverse-transcribe and amplify TCRβ CDR3 regions (**S1 Table**). The reaction was performed in a total of 25 μl of reaction mix in 1 well.

2) We performed multiplex PCR using the Multiplex PCR Kit (Qiagen) and multiple Vα and Vβ region primers and Cα and Cβ region primers (**S2 Table**) in a total 10 μl of reaction mix. To add specific barcodes for each TCR3 region, we added common sequences (lower case) to the 5’ ends of the primers.

3) We performed an eighth cycle of PCR using barcoding primers (**S3 Table**). The 3’ barcoding primers contained sequences that match the common sequences of the second amplification products, and the 5’ barcoding primers contained 8 nt of specific sequences (bold) The combination of the 5’ and 3’ barcoding primers was used to uniquely label each individual well (i.e., single T cells) in a 96-well plate.

4) We used the pooled PCR products of the third amplification for NGS with the MiSeq system (Illumina, San Diego, CA) to retrieve each T cell’s CDR3 sequences of TCRα and TCRβ.

### Rapid assembly of the TCR construct in the sleeping beauty transposon/transposase system

Individual CDR3 gene fragments generated by PCR contained a 15-bp overlap sequence at the 3’ end of each oligonucleotide. To assemble the 4 gene fragments, we mixed 50 ng of TRAV-TRBC2-pSB linear DNA, 9.4 ng of TRAC-TRBV linear DNA, 1.4 ng of TCRα CDR3, 1.4 ng of TCRβ CDR3, and 2x Gibson Assembly mix (New England Biolabs, Ipswich, MA) in a 0.2-mL PCR tube. After 1 hour of incubation at 50°C, 5 μL of the sample was used to chemically transform 25 μL of competent cells (*E*. *Cloni;* Lucigen, Middleton, WI). Colonies were selected and expanded in lysogeny broth containing kanamycin. SB DNA plasmids were isolated using the Miniprep kit or Midiprep kit (Qiagen), and coding sequences were verified by Sanger sequencing.

### Gene transduction

DNA plasmids were introduced into the Jurkat 76 cell line and its derivatives using Nucleofector II or 4D (Lonza). In brief, 1−2 x 10^6^ cells were resuspended in 100 μL of kit V buffer and DNA plasmid (or mRNA transcribed in vitro) and then cuvette-pulsed using program X-001 (Nucleofector II) or resuspended in SE buffer and cuvette-pulsed using program CL-120 (Nucleofector 4D). We used the same number of cells in 100 μL of buffer. The DNA plasmid ratio of SB transposon to SB transposase (SB11 or SB100x) was 3:1. After electroporation, cells were transferred to a well in a 12-well plate containing 3 mL of complete medium and maintained at 37°C in 5% CO_2_. After the SB system was used to transfect Jurkat 76 cells with the CD8 αβ heterodimer, CD8^+^ clones were isolated by limiting dilution. Enrichment of the cells transfected with the NR4A1 reporter construct was done by selection mediated by a neomycin analogue (G418; Sigma-Aldrich, St. Louis, MO). Resting CD8^+^ T cells and CD8^+^ T cells activated by stimulation with a CD3-specific mAb (clone OKT3, eBiosciences, San Diego, CA) and CD28-specific mAb (clone CD28.2; eBiosciences) for 48 hours were used for electroporation. Electroporation was done in Nucleofector 4D with P3 buffer using the FI-115 program for resting T cells and the EO-115 program for activated T cells.

### Reporter assay

Minor histocompatibility antigen HA2/HLA-A2−specific TCRαβs [20] and low- and high-affinity NY-ESO-1/HLA-A2−specific TCRαβs [21] were used for the reporter assay. TCRαβ-modified reporter cells were mixed with stimulator cells at a 1:1 ratio in 15-mL round-bottomed tubes or in 96-well U-bottomed plates. The tubes or plates were briefly spun at 300 x g for 3 minutes, and the cells were cultured at 37°C at 5% CO_2_. For CD3-mediated stimulation, the surface of 15-mL round-bottomed tubes or in 96-well U-bottomed plates was coated with 10 μg/mL OKT3 antibody for 3 hours at 37°C or overnight at 4°C and then washed 3 times with PBS. After 24, 48, 72, and 96 hours, the cells were stained with CD3-specific and CD8-specific mAbs for 15 minutes at 4°C. Data were acquired using a MACSQuant or iQue flow cytometer and analyzed using FlowJo version 10.1 or ForeCyt. Statistical significance was analyzed with the Student t-test in GraphPad Prism 7 (GraphPad Software, Inc. San Diego, CA).

### ^51^Chromium release assay

Target cells were labeled with 0.1 mCi of ^51^Cr (Perkin Elmer, Boston, MA) for 2 hours. The labeled cells were washed 3 times with ice-cold complete medium, diluted, and plated in 100 μL of complete medium in 96-well V-bottomed plates at 1 x 10^3^ cells/well. T cells were added at 100 μL/well at the indicated effector-to-target ratios, and the plates were spun (180 x g for 3 minutes without brake) to facilitate cell-to-cell contact. After a 4-hour incubation at 37°C in 5% CO_2_, 50 μL of the supernatants were counted on TopCount (Perkin Elmer). All assays were performed in triplicate. The percentage of specific lysis was calculated as follows: [(experimental cpm—spontaneous cpm) / (maximum cpm—spontaneous cpm)] × 100. Statistical significance was analyzed with the Student t-test in GraphPad Prism 7.

### In vivo analysis

Fifteen 10-week-old female NOD-scid IL2Rγ^null^ (NSG) mice (The Jackson Laboratory, Bar Harbor, ME) were injected with 5 x 10^5^ U266 cells modified with firefly luciferase as described previously [22] on day 0. On day 1, the mice received tail vein injections of 1 x 10^6^ T cells modified with HLA-A2−restricted NY-ESO-1−specific TCRαβs (1 x 10^6^ NY-ESO-1/HLA-A2 pentamer^+^ cells). Tumor expansion was periodically monitored using the Xenogen IVIS Spectrum in vivo imaging system (Caliper Life Sciences, Waltham, MA) 10 minutes after subcutaneous injection of 215 μg d-luciferin potassium salt (Caliper Life Sciences). Tumor flux (photons/second/cm^2^/steradian) was measured using Living Image software (version 2.50; Caliper Life Sciences). The mice were monitored daily for early signs of morbidity (ruffled fur, weakness, and hunched posture) throughout the course of the experiment. Moribund mice were immediately euthanized at presentation of hyperpnea, weight loss exceeding 20%, and hind limb paralysis.

### Endogenous HLA class I disruption from HEK293T cells and expression of HLA class I cloned by locus specific primers

CRISPR-Cas9 targeting consensus sequences (GGCTACTACAACCAGAGCG[AGG], [AGG]: PAM sequence) specific to classic HLA class I genes were generated in a DNA plasmid (px330; Addgene, Watertown, MA). DNA plasmids were transfected into HEK293T cells using polyethylenimine. HLA class I^neg^ HEK293T cells were isolated by negative selection using an LD column (Miltenyi Biotec) with APC-conjugated mAbs specific for HLA-A, -B, and -C (clone G46-2.6; BD Biosciences) and APC microbeads (Miltenyi Biotec). The sequences of the HLA cloning primers were as follows (lower case indicates the overlap sequence for Gibson assembly; upper case indicates the locus-specific sequence):

HLA-A forward: cgcagtcagtgctctagagctagcg GATTCTCCCCAGACSCCGAGG
HLA-A reverse: gtaatccagaggttgattgtcgacgc ACAAGGCAGCTGTCTCACA
HLA-B forward: cgcagtcagtgctctagagctagcg CACCCGGACTCARARTCTCCT
HLA-B reverse: gtaatccagaggttgattgtcgacgc CCTTTTCAAGCTGTGAGAG
HLA-C forward: cgcagtcagtgctctagagctagcg TTCTCCCCAGASGCCGAGATG
HLA-C reverse: gtaatccagaggttgattgtcgacgc GTCTCAGGCTTTACAAGYGA

We amplified 50 ng of cDNA by PCR using KOD Xtreme DNA polymerase (EMD Millipore, Burlington, MA) according to the manufacturer’s instructions. The PCR product was used for Gibson assembly with the pCDH (EF1) MCS (PGK) Puro lentivirus plasmid (SBI System Biosciences, Palo Alto, CA) linearized by restriction enzyme (*EcoRV* and *NotI*) and subsequent mung bean nuclease (New England Biolabs) treatment.

The pCDH plasmid encoding HLA, along with pMD2.VSVG and psPAX2 (Addgene), was transfected into HEK293T cells by polyethylenimine. Two days after transfection, half of the culture medium was removed and stored at -80°C as lentivirus particles. The remaining medium was amended with 8 μg/mL polybrene and cultured overnight. The next morning, the medium was replaced with Iscove’s modified Dulbecco’s medium supplemented with 10% FBS and further cultured at 37°C in 5% CO_2_.

## Results

### Generation of a DNA library for the rapid assembly of TCRαβ genes in the SB system

**Fig 1:**
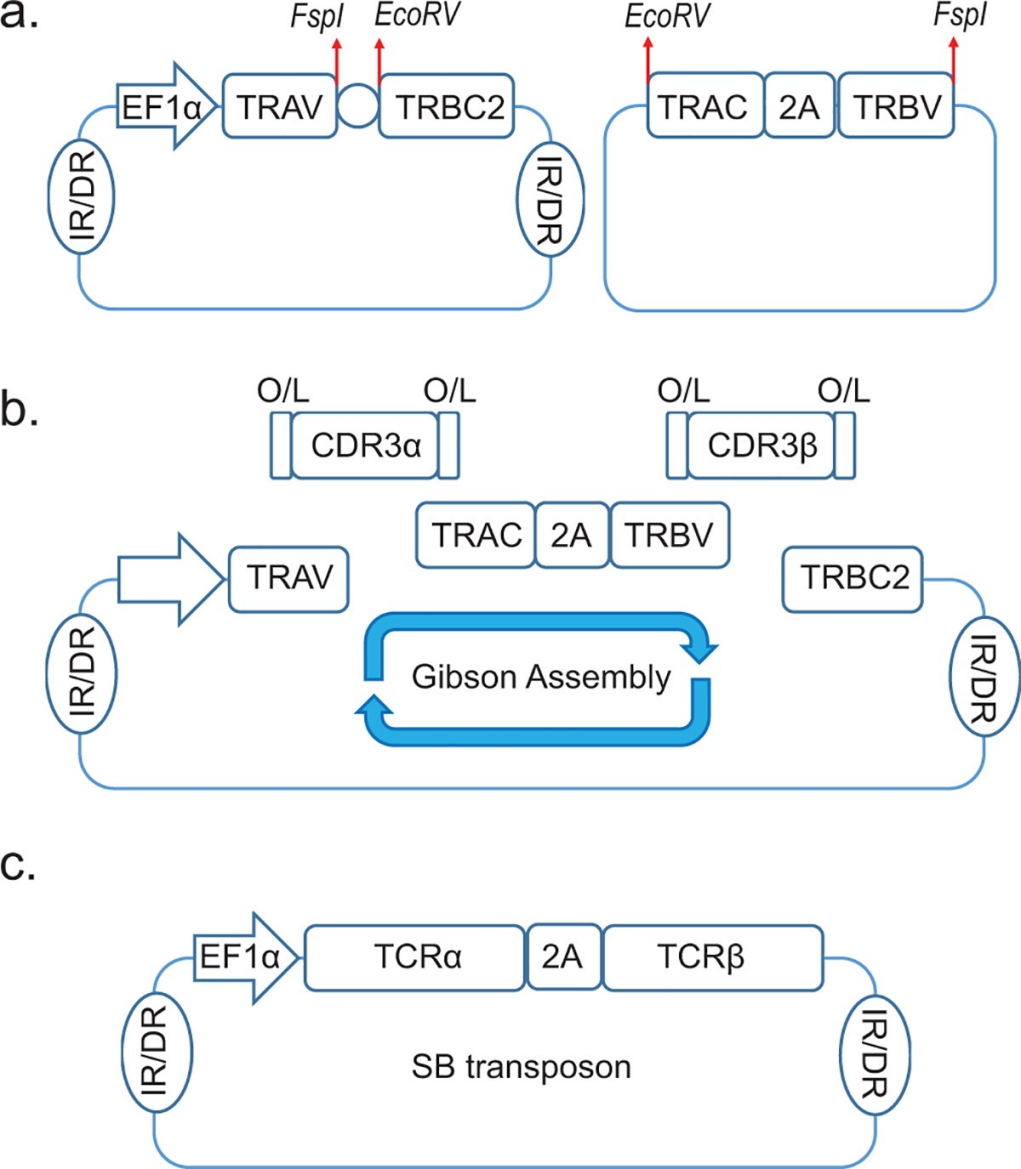
TCR plasmid libraries used for the rapid construction of the TCRαβ gene in the Sleeping Beauty system. **a.** The plasmid maps for the TRAV-TRBC2 (left) and TRAC-TRBV (right) plasmid libraries. We prepared and stored 45 TRAV-TRBC2 and 48 TRAC-TRBV plasmid libraries for the rapid construction of the TCRαβ gene. IR/DR, inverted repeats/direct repeats (a transposase binding site). **b.** Linearized TRAV-TRBC2 and TRAC-TRBV plasmids, along with the TCRα chain CDR3 sequence (CDR3α) and TCRβ chain CDR3 sequence (CDR3β), were joined using Gibson assembly. O/L, overlap sequence (necessary for Gibson assembly). **c.** Isothermal 1-hour Gibson assembly resulted in a single SB DNA plasmid encoding the TCRαβ gene.

scRNA-seq can identify hundreds of unique paired CDR3 sequences of TCRα and TCRβ chains from single T cells. We developed a method to clone and express large numbers of TCRs in a high-throughput manner by generating a TCR library in SB-derived DNA transposons (plasmids). The SB system, which is being evaluated in clinical trials, has advantages over clinical-grade virus-based systems in that it is rapid, scalable, and inexpensive [23, 24]. The library consists of 48 TCRα sequences as SB plasmids, each assembled from an individual TCR alpha variable (TRAV) gene paired with a consensus TCR beta constant 2 (TRBC2) gene sequence. Similarly, a library of 59 TCRβ sequences encoding consensus TCR alpha constant (TRAC) genes linked with 59 unique TCR beta variable (TRBV) genes by the furin-SGSG-P2A ribosome-skipping sequence was constructed (**Fig 1A and 1B and S1 Fig**). These DNA plasmids were designed to be linearized by 2 blunt-end restriction enzymes (*EcoR*V and *Fsp*I). CDR3 sequences retrieved from NGS were generated by the PCR of 2 single-strand DNAs with a 15-bp overlap (**S2 Fig**). The complete SB DNA plasmid was created by using Gibson assembly [25] to join 4 DNA fragments, including an approximately 130-bp TCRα CDR3 gene fragment, an approximately 130-bp TCRβ CDR3 gene fragment, a linearized TRAC-TRBV fragment, and a linearized TRAV-TRBC2 fragment (**Fig 1C**). Each resultant SB transposon encodes a unique assembled TCRαβ gene and can be integrated into primary T cells or cell lines with a SB transposase.

### Tetramer-guided isolation of CMVpp65/HLA-A2−specific TCR by paired single-cell TCRαβ cloning and rapid assembly of TCRαβ genes

To test our approach to constructing the TCRαβ library, we used tetramer-guided single-cell sorting to isolate T cells expressing CMVpp65/HLA-A2−specific TCRαβs from HLA-A2^+^ PBMCs. We performed multiplex PCR with barcoded primer sets [19] followed by NGS with the Illumina MiSeq system to determine CDR3 sequences (**S3 Fig**). We identified 10 unique TCRαβ CMVpp65/HLA-A2−specific paired CDR3 sequences (**Table 1**), which we used to generate 10 full-length TCRαβ genes as SB transposons in 3 days. Thus, Gibson assembly of SB-derived DNA plasmids encoding individual TCR genes proved to be an efficient and robust method for rapidly generating TCRαβ constructs from CDR3 sequences obtained from scRNA-seq data.

**Table 1:** Unique CMV (CMVpp65/HLA-A2: NLVPMVATV) specific TCRαβ sequences obtained from CMV seropositive donor.

UCN	CDR3			TCR assembly
CMV1	TRAV3*01	TRAJ26*01		Success
	TGCGCTGTATACTATGGTCAGAATTTTGTCTTT			
	CAVYYGQNFVF			
	TRBV28*01	TRBD1*01	TRBJ1-1*01	
	TGCGCCAGCAGTAACCAGGGGTACACTGAAGCTTTCTTT			
	CASSNQGYTEAFF			
CMV2	TRAV*3*01	TRAJ26*01		Success
	TGCGCTGACTACTATGGTCAGAATTTTGTCTTT			
	CADYYGQNFVF			
	TRBV28*01	TRBD1*01	TRBJ1-1*01	
	TGCGCCAGCAGTTACCAGGGTTACACTGAAGCTTTCTTT			
	CASSYQGYTEAFF			
CMV3	TRAV25*01	TRAJ39*01		Success
	TGCGCCACTAATGCAGGCAACATGCTCACCTTT			
	CATHAGNMLTF			
	TRBV28*01	TRBD1*01	TRBJ2-3*01	
	TGCGCCAGCAGTTTCTTGACAGGGGTGGGGGATACGCAGTATTTT			
	CASSFLTGVGDTQYF			
CMV4	TRAV3*01	TRAJ31*01		Success
	TGCGCTGTGAGAGACATAAATGCCAGACTCATGTTT			
	CAVRDINARLMF			
	TRBV12-3*01	TRBD	TRBJ1-1*01	
	TGCGCCAGCAGTTCAGTGAACGAAGCTTTCTTT			
	CASSSVNEAFF			
CMV5	TRAV3*01	TRAJ31*01		Success
	TGCGCTGTGAGAGACGTGAATGCCAGACTCATGTTT			
	CAVRDVNARLMF			
	TRBV12-3*01	TRBD	TRBJ1-1*01	
	TGCGCCAGCAGTTCGGTCAATGAAGCTTTCTTT			
	CASSSVNEAFF			
CMV6	TRAV35*01	TRAJ50*01		Success
	TGCGCTGGCCCAACGAAAACCTCCTACGACAAGGTGATATTT			
	CAGPTKTSYDKVIF			
	TRBV12-3*01	TRBD	TRBJ1-2*01	
	TGCGCCAGCAGTTCGGCCTACTATGGCTACACCTTC			
	CASSSAYYGYTF			
CMV7	TRAV26-2*01	TRAJ49*01		Success
	TGCATCCTGAGTGGCTCAGAGGGCCAGTTCTATTTT			
	CILSGSEGQFYF			
	TRBV29-1*01	TRBD	TRBJ2-2*01	
	TGCAGCGTCCACTCTTATGGGGACACCGGGGAGCTGTTTTTT			
	CSVHSYGDTGELFF			
CMV8	TRAV26-2*01	TRAJ47*01		Success
	TGCATCCTGAGACAGGAATATGGAAACAAACTGGTCTTT			
	CILRQEYGNKLVF			
	TRBV16*01	TRBD	TRBJ2-7*01	
	TGCGCCAGCAGCCAAGGGGAGCTAGGGACTAGCGGGAGCCACGAGCAGTACTTC			
	CASSQGELGTSGSHEQYF			
CMV9	TRAV3*01	TRAJ47*01		Success
	TGCGCTGTGGAATATGGAAACAAACTGGTCTTT			
	CAVEYGNKLVF			
	TRBV27*01	TRBD	TRBJ2-7*01	
	TGCGCCAGCAGCCCCGTAGCGGGAGCCCCCCACGAGCAGTACTTC			
	CASSPVAGAPHEQYF			
CMV10	TRAV24*01	TRAJ43*01		Success
	TGCGCCTTCCCGTACAATAACAATGACATGCGCTTT			
	CAFPYNNNDMRF			
	TRBV27*01	TRBD2*02	TRBJ1-1*01	
	TGCGCCAGCAGTTTAGAGGGTTACACTGAAGCTTTCTTT			
	CASSLEGYTEAFF			

### Generation of reporter cell lines for measuring the antigen specificity of introduced TCRs

**Fig 2:**
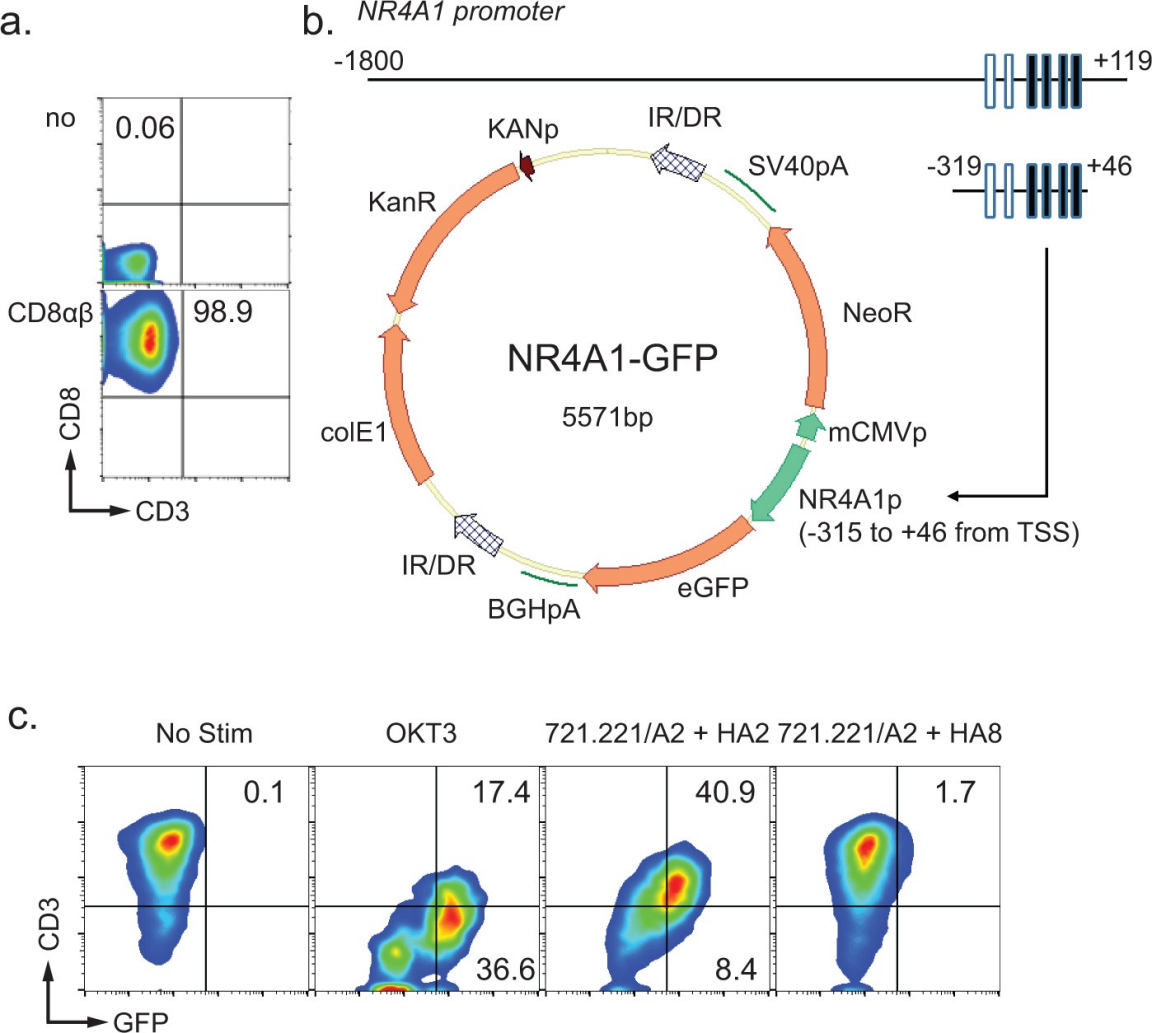
Generation of the JRFTCR. **a.** Introduction of the CD8αβ heterodimer. The SB system was used to introduce CD8αβ into the Jurkat 76 cell line. Data are the CD8 expression levels in isolated clones. **b.** The top panel shows the NR4A1 promoter. Closed boxes represent cAMP response element binding protein (CREB) response elements; open boxes represent myocyte enhancer factor-2 (MEF2) response elements. The bottom panel shows the plasmid map for the reporter construct. An SB DNA plasmid carrying a bi-directional promoter (NR4A1 and a minimal CMV promoter) was generated as shown. IR/DR, inverted repeat; NeoR, neomycin resistant gene; eGFP, enhanced GFP. **c.** Reporter assay using JRFTCRs transduced with HA2/HLA-A2−specific TCRαβ genes. The JRFTCRs were stimulated with HLA-A2−transduced 721.221 cells pulsed with either a cognate HA2 peptide (YIGEVLVSV) or an irrelevant HA8 peptide (RTLDKVLEV) [27]. Numbers indicate the percentages of GFP^+^cells.

To enable the measurement of the antigen specificity of introduced TCRαβs, we used the Jurkat 76 cell line, which is deficient in both TCRα and TCRβ [20]. First, we modified Jurkat 76 cells to express CD8αβ heterodimer to stabilize their expression of TCRs with HLA class I molecules (**Fig 2A**). Next, we cloned NR4A1 promoter sequences from healthy donor T-cell genomic DNA to express EGFP under the control of this TCR-dependent activation promoter (**Fig 2B**). We tested NR4A1 promoter sequences of varying lengths, including those with regions for binding the transcription factors CREB (cAMP response element binding protein) and MEF2 (myocyte enhancer factor-2) [26]. Constructs of 1,919 bp (from -1,800 to +119 of the transcription start site) and 365 bp (from -319 to +46 of the transcription start site) (**Fig 2B and S4 Fig**) were tested using HA2/HLA-A2 minor histocompatibility antigen−specific TCRαβs [20]. The minimal 365-bp promoter sequence was sufficient to induce GFP expression when the introduced TCRαβ was stimulated with a cognate antigen and OKT3 antibody (**Fig 2C**). The level of GFP expression induced by the NR4A1 minimal promoter element was sufficient to be detected by flow cytometry and fluorescence microscopy at the single-cell level (**S5 Fig**). On the basis of these data, we selected the 365-bp NR4A1 promoter−driven GFP as our reporter construct and designated our reporter cell clone Jurkat reporter for TCR (JRFTCR).

### JRFTCR GFP expression reflects TCR avidity and function in vitro and in vivo

**Fig 3:**
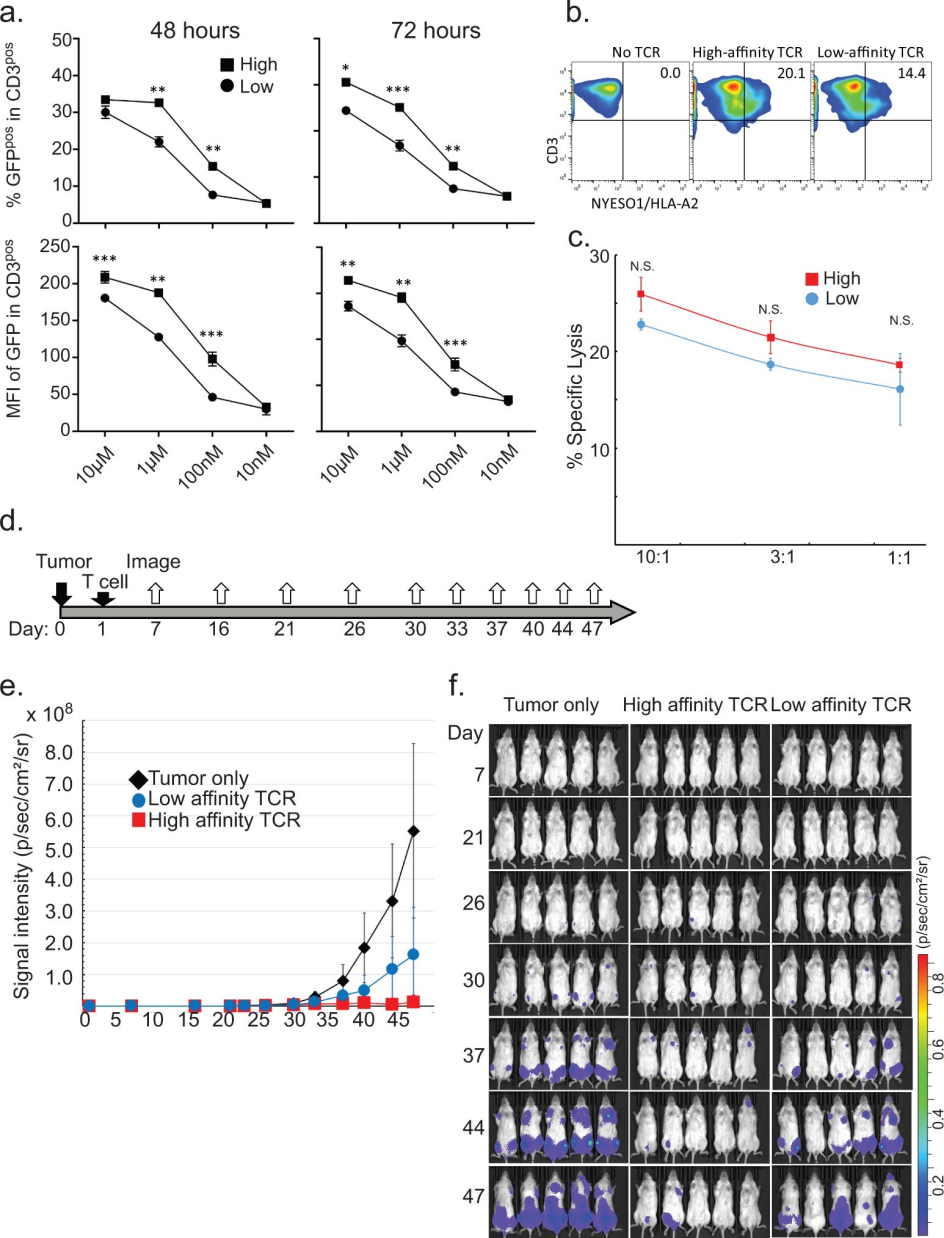
Comparison of high- and low-affinity NY-ESO-1/HLA-A2−specific TCR. **a.** Our reporter assay revealed the GFP expression of each JRFTCR transduced with NY-ESO-1/HLA-A2−specific TCR and then stimulated with HLA-A2−transduced 721.221 cells pulsed with different amounts of the NY-ESO-1/HLA-A2 peptide (SLLMWITQV). The top panels show the percentages of CD3^+^ cells (cells expressing NY-ESO-1/HLA-A2−specific TCR) with GFP expression. The bottom panels show the mean fluorescence intensity (MFI) of GFP in the CD3^+^ population. Error bars indicate standard deviations. *p < 0.01; **p < 0.005; ***p < 0.001. **b.** Multimer staining of T cells transfected with NY-ESO-1/HLA-A2−specific TCR. The numbers represent the percentages of CD3^+^ cells expressing NY-ESO-1/HLA-A2−specific TCR. The data were acquired 2 days after electroporation. **c.** ^51^Cr release assay revealed the activity of T cells transduced with NY-ESO-1/HLA-A2−specific TCR against HLA-A2^+^ NY-ESO-1^+^ U266 cells. Error bars indicate standard deviations. N.S., not significant (p > 0.01). **d.** Timeline of the xenograft experiment. **e.** Luciferase-transduced U266 xenografts in NSG mice were monitored periodically using the Xenogen IVIS Spectrum system. **f.** Tumor xenografts in NSG mice.

To evaluate the kinetics of GFP expression in JRFTCRs after antigen stimulation, we compared high- and low-affinity NY-ESO-1/HLA-A2 TCRαβs [21]. JRFTCRs expressing NY-ESO-1/HLA-A2−specific TCRαβ were stimulated with HLA-A2 transduced 721.221 cells pulsed with graded levels of NY-ESO-1−derived peptide and were serially assessed for GFP expression and intensity (**Fig 3A and S6 Fig**). After 24 hours of co-culture with the antigen-presenting cells, JRFTCRs expressing NY-ESO-1/HLA-A2−specific TCRαβ showed GFP expression. The CD3 expression of TCR^+^ JRFTCRs stimulated through TCR or the CD3 complex was significantly lower than that of unstimulated TCR^+^ JRFTCRs or TCR^+^ JRFTCRs stimulated with irrelevant antigens. CD3 expression usually returned within 5 days after stimulation (**S7 Fig**). The percentage of GFP^+^ reporter cells and the mean fluorescence intensity of GFP expression were correlated with TCR affinity. JRFTCRs transduced with high-affinity NY-ESO-1/HLA-A2−specific TCRαβ (1 μM−100 nM) showed significantly higher GFP expression and mean fluorescence intensity than those transduced with low-affinity NY-ESO-1/HLA-A2−specific TCRαβ did. When the amount of peptide was decreased to less than 10 nM, GFP expression was abolished, revealing the assay’s limit of detection.

Next, we tested whether the observed differences in GFP expression and intensity were correlated with the *in vitro* and *in vivo* function of peripheral blood T cells genetically modified to express high- or low-affinity NY-ESO-1/HLA-A2−specific TCRαβ. T cells were stimulated with OKT3 and an anti-CD28 mAb for 2 days and then nucleofected with the SB DNA plasmids. NY-ESO-1/HLA-A2−specific TCR expression was present on 14% and 20% of the T cells transfected with low- or high-affinity NY-ESO-1/HLA-A2−specific TCR, respectively (**Fig 3B**).

U266, a multiple myeloma cell line that co-expresses HLA-A2 and NY-ESO-1, was used as a target for primary T cells expressing NY-ESO-1/HLA-A2−specific TCR. We performed a ^51^Cr release killing assay and an *in vivo* anti-tumor assay using xenograft mice. High-affinity TCRαβ^+^ T cells tended to lyse the target U266 cells more efficiently than low-affinity TCRαβ^+^ T cells did, especially at an increased effector-to-target ratio, although this difference was not statistically significant (**Fig 3C**). Among mice injected with luciferase-transduced U266 cells, those left untreated showed tumor engraftment as early as day 30; by day 47, these mice had to be euthanized owing to the rapidly growing tumors. Compared with these control mice, the mice that received TCRαβ^+^ T cells on day 1 had delayed tumor engraftment. High-affinity TCRαβ^+^ T cells suppressed tumor engraftment better than low-affinity TCRαβ^+^ T cells did. The therapeutic efficacy of the TCRαβ-expressing T cells against the U266 cells was in good agreement with the *in vitro* data (**Fig 3A and 3D–3F**).

### Use of locus-specific primer pairs for rapid cloning of HLA class I and II in HLA^neg^ HEK293T cells

**Fig 4:**
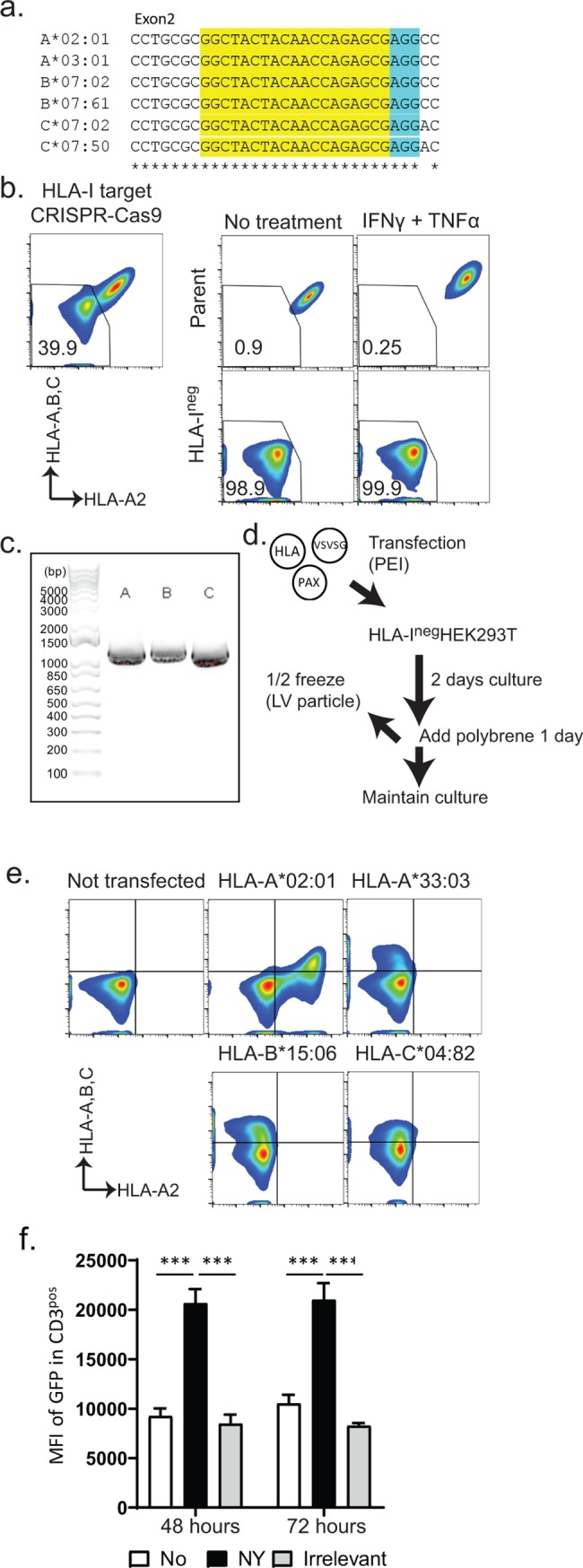
HLA cloning and expression in HLA class I−disrupted HEK293T cells. **a.** Guide RNA was designed to bind consensus sequences of HLA class I (HLA-A, -B, and -C) expressed in HEK293T cells. The target sequence is highlighted in yellow; the PAM sequence for SpCas9 nuclease is highlighted in blue. **b.** Elimination of HLA class I expression in HEK293T cells. Data in the left panel are from HEK293T cells transfected with a single DNA plasmid encoding guide RNA and Cas9. Three days after transfection, the HEK293T cells were stained with mAbs against HLA-A2 and HLA-A, -B, and -C. Numbers indicate the percentages of HLA class I−negative HEK293T cells. Data in the right panel are from cells cultured with 600 IU/mL interferon-γ (IFNγ) and 10 ng/mL tumor necrosis factor α (TNFα) for 2 days. **c.** Representative agarose gel image of HLA class I cloning from cDNA generated from PBMCs. **d.** Transfection of the HLA-encoding lentivirus plasmid along with helper plasmids into HEK293T cells and subsequent culture to generate HEK293T cells expressing the HLA of interest. **e.** Representative expression of cloned HLA class I on HEK293T cells. Each number represents the expression of the introduced HLA on HLA class I−negative HEK293T cells. **f.** Mean fluorescence intensity (MFI) of JRFTCRs transfected with NY-ESO-1/HLA-A2−specific TCR and then stimulated with peptide-pulsed or irrelevant peptide−pulsed HEK293T cells. *p < 0.01; **p < 0.005; ***p < 0.001.

By stimulating them with antigen-presenting cells, JRFTCRs can be used as an antigen-discovery platform. To be used in a high-throughput system, antigen-presenting cells must have a high transfection efficiency for the introduction of both HLA and potential tumor antigens and should be adherent cells, so as to minimize contamination of the flow-cytometric evaluation in the reporter assay. To establish such stimulator cells for this assay, we generated a system to rapidly express a patient’s HLA molecules on HEK293T cells whose endogenous HLA class I expression has been disrupted by CRISPR-Cas9 gene editing. Other investigators have used the CRISPR-Cas9 system to target β_2_M to globally disrupt HLA class I expression. However, disruption of this locus adversely affects the expression of introduced HLA class I constructs, unless those constructs comprise a single-chain HLA molecule containing β_2_M and an antigen-derived peptide [28]. To avoid this complexity, we targeted a sequence that is common to all HLA class I genes expressed on HEK293T cells (HLA-A*02:01, A*03:01, HLA-B*07:02, B*07:61, HLA-C*07:02, and C*07:50; **Fig 4A**). Single transduction of DNA plasmid encoding the guide RNA and wild type *S*. *pyrogenes* Cas9 (SpCas9) nuclease eliminated HLA class I expression in up to 40% of HEK293T cells. By negative selection and single-cell cloning of the HLA^neg^ population, we established HLA class I^neg^ HEK293T cells, which do not express HLA class I, even after they have been cultured with interferon-γ and tumor necrosis factor-α for 2 days (**Fig 4B**).

Next, we used locus-specific universal PCR-based cloning and subsequent Gibson assembly to generate lentivirus vector plasmids for inducing the expression of HLA molecules. Primers were designed as described previously [29] and modified to amplify either coding sequences or coding sequences plus short 5’UTR sequences, depending on the availability of the consensus sequence in each HLA class I locus. The HLA class I coding sequences were cloned from cDNA (**Fig 4C**). The HLA-A, -B, -C alleles were authenticated through direct use of the PCR product for Gibson assembly and by subsequent Sanger sequencing. Transfecting HLA class-I^neg^ HEK293T cells with the HLA-coding lentivirus plasmid and helper plasmids produced lentivirus particles in the culture media. Briefly culturing the transfected cells with polybrene generated HEK293T cells that had sustained expression of a single HLA class I molecule (**Fig 4D and 4E**). These cells could stimulate TCR-transfected JRFTCRs in 96-well plates (**Fig 4F**). Using this system, we also designed sets of universal primers specific for HLA class II and efficiently amplified HLA-DRA, -DRB, -DQA, -DQB, -DPA, and -DPB (**S8 Fig**).

## Discussion

We established a rapid, robust, high-throughput platform for generating and expressing paired TCRα and TCRβ identified by single-cell TCR sequencing. scRNA-seq technology has immensely improved the high-throughput characterization of TILs and other disease-related T cells [18]. The combined evaluation of transcriptome and paired TCR (CDR3) sequencing can provide in-depth information on hundreds to thousands of T cells of interest [30–33]. Typically, the paired CDR3 sequences obtained from these assays have been used to evaluate T-cell clonality. Expanding such knowledge to assess infiltrating T cells’ antigen specificity would be advantageous in designing and improving antigen-specific tumor immunotherapy and minimizing the potential toxicities effected by the binding promiscuity of TCR. To achieve this, we established a system for rapidly assembling TCRαβ genes constructed as SB transposons. Once the paired CDR3 sequences were obtained, TCRαβ library was constructed in only a few days. Others have assembled a library of TCRαβ DNA species using Golden Gate cloning and subsequent cloning into a lentivirus vector [34]. Our Gibson assembly method typically takes only 1 hour with isothermal incubation, making assembly relatively quick and easy. Moreover, our SB system has an added advantage over virus-based systems, as once the DNA plasmid has been assembled and generated, it can be used with a transposase plasmid to generate TCRαβ-transduced cells. This rapid assembly of TCRαβ genes has the potential for clinical translation. Accumulating evidence suggests that immune checkpoint blockade cannot sustain reinvigorated tumor-specific T cells [35]. A better way to target solid tumors may be the *in vitro* generation and subsequent administration of young T cells that have been genetically modified to express tumor-specific TCRαβs [36]. The rapid generation of hundreds of tumor-specific TCRαβs is an important step towards achieving this immunotherapy goal.

NGS of TCR revealed that 14% of T cells have 2 functional (productive) TCRα transcripts [19]. In the present study, we found that of 86 single T cells, 2 (2.3%) expressed 2 functional TCRαs and 1 (1.1%) expressed 2 functional TCRβs. These T cells’ antigen specificity needs to be determined by making 4 possible TCR heterodimers. However, considering the small percentage of those T cells and our very rapid TCR gene assembly method, the evaluation of the antigen specificity of those T cells would be feasible.

In other TCR reporter systems, luciferase activity [37] or cytokine production [38] are used to measure signaling. In one fluorescence-based reporter system [39, 40], the use of flow cytometry or fluorescence microscopy enabled the direct detection of reporter cells responding to antigen-mediated stimulation. This detection facilitated the fluorescence-based single-cell isolation of antigen-specific TCRs. In general, researchers have used nuclear factor of activated T cells (NFAT) inducible elements to generate reporter cell lines to conditionally measure antigen-specific activation through TCRαβs [34, 41, 42]. However, NFAT expression does not rely on only the TCR signal; it is also influenced by cytokine stimulation [43, 44], which increases the signal-to-noise ratio. Furthermore, NFAT elements alone do not induce strong downstream gene expression. To maximize the detection of induced gene expression, researchers have combined the minimal CMV or IL-2 promoter with multiple repeats of NFAT elements and have deployed luciferase expression as a reporter [37, 45]. As an alternative to NFAT, we chose NR4A1, a nuclear orphan receptor whose expression is induced by the activation-induced membrane depolarization and subsequent increase of intracellular Ca^2+^ [46]. NR4A1 was initially identified as a mediator of apoptosis in response to TCR engagement in immature T cells [47]. NR4A1 also appears to act as a fate-determination molecule in T-cell differentiation [48–50]. Recent mouse experiments revealed the rapid and robust expression of NR4A1 in T cells and even higher expression of the protein in regulatory T cells. Other investigators showed that the genetic insertion of a GFP reporter downstream of the NR4A1 promoter induces fluorescence after TCR-mediated stimulation [51]. Furthermore, we simplified the NR4A1 construct by using a 365-bp sequence as a short regulatory element within the NR4A1 promoter, which was sufficient and necessary to drive conditional GFP expression. In a previous study using a different mouse model, NR4A1 promoter−mediated GFP expression peaked at 24 hours [52]. In the present study, using the short NR4A1 promoter, we found that GFP expression increased for 72 hours, which may have been due to continuous TCR stimulation. We found this NR4A1 promoter system to be superior to NFAT-based reporter systems, and we were able to detect GFP expression at the single-cell level. Moreover, we found that the GFP expression level correlated well with TCRαβ affinity and with the functions of the identified TCR in primary T cells.

T-cell specificity screening is a time-consuming and laborious process. Computer algorithms have been used to predict immunogenic neoantigens from tumor whole-exome sequencing and transcriptome sequencing. Refining these algorithms will enable them to identify neoantigens more precisely. [Au: A transition is needed here, I think.] By stimulating them with peptide-pulsed cells or subjecting them to peptide/HLA multimer staining, our JRFTCRs can be used to evaluate whether cloned TCRs recognize predicted neo-epitopes. However, we can also use a flow cytometry−based approach to interrogate minigene constructs in a high-throughput manner [53]. The use of stimulator cells pulsed with synthetic peptides may not reflect intracellular protein degradation and HLA loading machinery. Instead, a plasmid-encoding minigene can be used, as the presentation of the neoantigen as a peptide-HLA complex on the cell surface requires the intracellular processing of the translated peptide. To perform this screening, we used HEK293T cells as founder antigen-presenting cells, given the easy maintenance and efficient expression of the introduced DNA plasmids. Because the neoantigens are presented as peptide-HLA complexes, the promiscuous binding of endogenously expressed HLA class I to the introduced TCRαβs would increase the background noise. Accumulating evidence suggests that TCRαβs promiscuously bind to non-cognate peptide-HLA complexes [54, 55]. To eliminate this background stimulation in TCRαβ^+^ JRFTCR, we established HLA class I^neg^ HEK293T cells. Combined with our rapid HLA cloning system, these edited HEK293 T cells can be used for screening.

Because they do not express endogenous TCRα or TCRβ chains, our JRFTCRs can be used to measure the specificity of the introduced TCR specifically. However, the introduction of tumor-specific TCRαβs into T cells may induce unintended specificity mediated through chimeric TCRαβ heterodimers owing to the 2 TCRα and 2 TCRβ transcripts in single T cells [56–58]. This may also affect the functions of TCR^+^ T cells owing to competition for the CD3 complex [59]. Those problems may be solved by using gene editing technologies to eliminate endogenous TCR expression [60]. In addition, our platform allows the targeted introduction of TCR constructs into TRAC and TRBC genes, which may enhance current TCR^+^ T-cell therapy while preventing unintended immune reactions [61].

**Fig 5:**
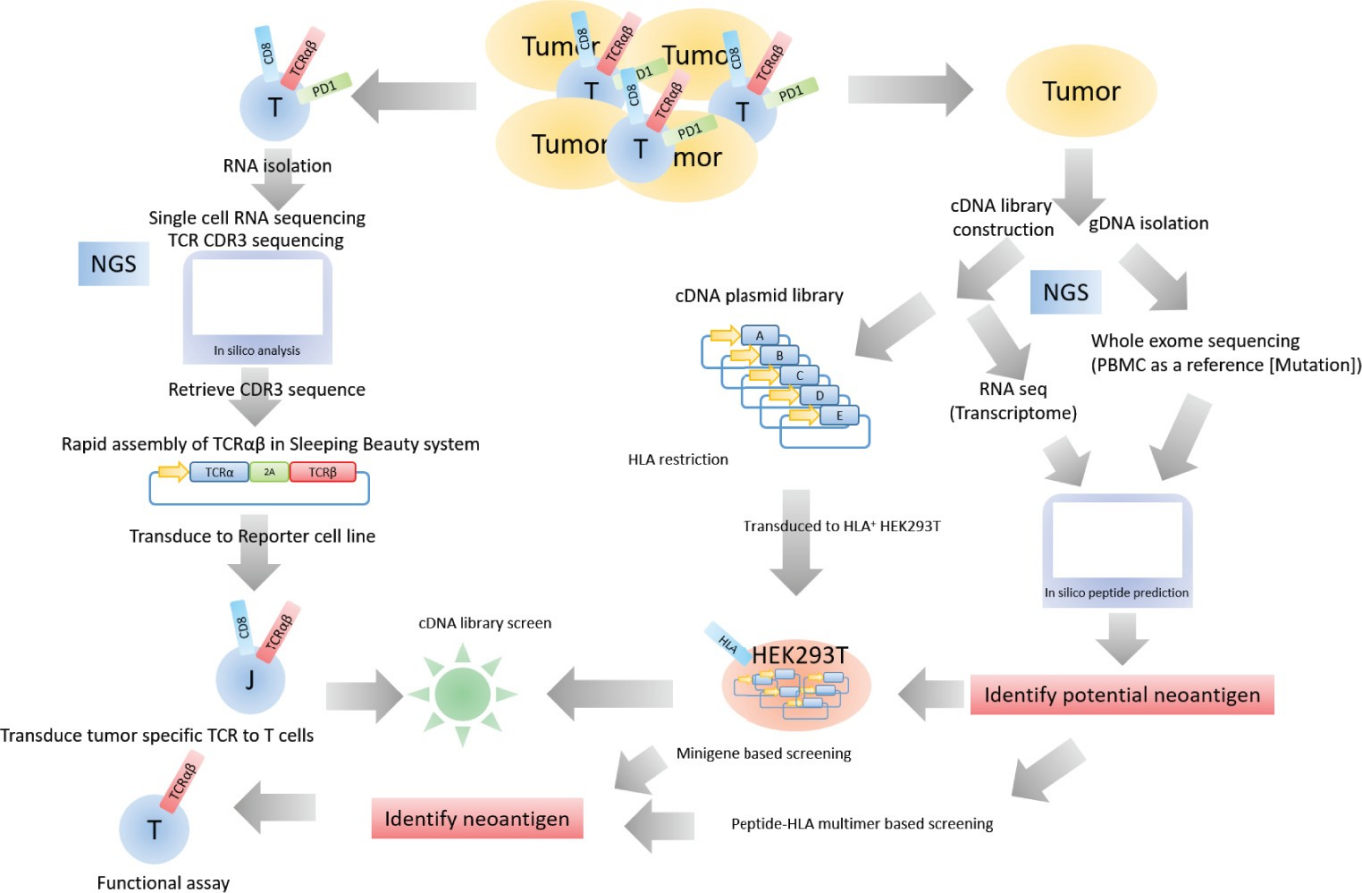
Potential application of the platform to identify tumor-specific TCRs and their cognate antigens.

In summary, we established a platform for cloning and determining the specificity of TCRαβs identified by scRNA-seq of single tumor-infiltrating T cells. This system is applicable to current efforts to evaluate the T-cell transcriptome, correlate with TCRαβ sequence, and discover the antigen landscape of TILs (**Fig 5**).

## Supporting information

S1 Fig Representative sequences of TCR plasmid library. (DOCX)

S2 Fig Sequences to be added to the 5’ and 3’ ends of CDR3 fragments. (DOCX)

S3 Fig Isolation of antigen-specific T cells and paired CDR3 sequencing of single antigen-specific T cells. (DOCX)

S4 Fig Sequences for the 2 NR4A1 promoters. (DOCX)

S5 Fig Fluorescence microscopy images of JRFTCRs stimulated with cognate antigen. (DOCX)

S6 Fig Reporter assay comparing JRFTCRs transduced with high- or low-affinity NY-ESO-1/HLA-A2−specific TCRαβs. (DOCX)

S7 Fig CD3 expression of TCR-transfected JRFTCRs after antigen-specific stimulation. (DOCX)

S8 Fig PCR cloning of HLA class II using a universal primer pair for each HLA class II locus. (DOCX)

S1 Table Primers for the initial amplification of the CDR3 regions of TCRα and TCRβ. (DOCX)

S2 Table Barcoded primers for the CDR3 regions of TCRα and TCRβ. (DOCX)

S3 Table 5’ and 3’ barcoding primers for NGS of the CDR3 regions of TCRα and TCRβ. (DOCX)

S1 Appendix The ARRIVE guidelines checklist. (PDF)

S2 Appendix Raw Gel image for Fig 4C and S8 Fig. (PDF)

## References

[1] Fridman WH, Pages F, Sautes-Fridman C, Galon J. The immune contexture in human tumours: impact on clinical outcome. Nature reviews Cancer. 2012;12(4):298–306. Epub 2012/03/16. 10.1038/nrc3245 . 22419253

[2] Galon J, Angell HK, Bedognetti D, Marincola FM. The continuum of cancer immunosurveillance: prognostic, predictive, and mechanistic signatures. Immunity. 2013;39(1):11–26. Epub 2013/07/31. 10.1016/j.immuni.2013.07.008 . 23890060

[3] Gentles AJ, Newman AM, Liu CL, Bratman SV, Feng W, Kim D, et al The prognostic landscape of genes and infiltrating immune cells across human cancers. Nature medicine. 2015;21(8):938–45. Epub 2015/07/21. 10.1038/nm.3909 26193342 PMC4852857

[4] Fridman WH, Zitvogel L, Sautes-Fridman C, Kroemer G. The immune contexture in cancer prognosis and treatment. Nature reviews Clinical oncology. 2017 Epub 2017/07/26. 10.1038/nrclinonc.2017.101 . 28741618

[5] Huang AC, Postow MA, Orlowski RJ, Mick R, Bengsch B, Manne S, et al T-cell invigoration to tumour burden ratio associated with anti-PD-1 response. Nature. 2017;545(7652):60–5. Epub 2017/04/12. 10.1038/nature22079 . 28397821 PMC5554367

[6] Lennerz V, Fatho M, Gentilini C, Frye RA, Lifke A, Ferel D, et al The response of autologous T cells to a human melanoma is dominated by mutated neoantigens. Proceedings of the National Academy of Sciences of the United States of America. 2005;102(44):16013–8. Epub 2005/10/26. 10.1073/pnas.0500090102 16247014 PMC1266037

[7] Rizvi NA, Hellmann MD, Snyder A, Kvistborg P, Makarov V, Havel JJ, et al Cancer immunology. Mutational landscape determines sensitivity to PD-1 blockade in non-small cell lung cancer. Science. 2015;348(6230):124–8. Epub 2015/03/15. 10.1126/science.aaa1348 25765070 PMC4993154

[8] Chabanon RM, Pedrero M, Lefebvre C, Marabelle A, Soria JC, Postel-Vinay S. Mutational Landscape and Sensitivity to Immune Checkpoint Blockers. Clinical cancer research: an official journal of the American Association for Cancer Research. 2016;22(17):4309–21. Epub 2016/07/09. 10.1158/1078-0432.CCR-16-0903 . 27390348

[9] Giannakis M, Mu XJ, Shukla SA, Qian ZR, Cohen O, Nishihara R, et al Genomic Correlates of Immune-Cell Infiltrates in Colorectal Carcinoma. Cell reports. 2016;15:857–65. Epub 2016/05/07. 10.1016/j.celrep.2016.03.075 27149842 PMC4850357

[10] Charoentong P, Finotello F, Angelova M, Mayer C, Efremova M, Rieder D, et al Pan-cancer Immunogenomic Analyses Reveal Genotype-Immunophenotype Relationships and Predictors of Response to Checkpoint Blockade. Cell reports. 2017;18(1):248–62. Epub 2017/01/05. 10.1016/j.celrep.2016.12.019 . 28052254

[11] Gros A, Robbins PF, Yao X, Li YF, Turcotte S, Tran E, et al PD-1 identifies the patient-specific CD8(+) tumor-reactive repertoire infiltrating human tumors. The Journal of clinical investigation. 2014;124(5):2246–59. Epub 2014/03/29. 10.1172/JCI73639 24667641 PMC4001555

[12] Fernandez-Poma SM, Salas-Benito D, Lozano T, Casares N, Riezu-Boj JI, Mancheno U, et al Expansion of Tumor-Infiltrating CD8+ T cells Expressing PD-1 Improves the Efficacy of Adoptive T-cell Therapy. Cancer research. 2017;77(13):3672–84. Epub 2017/05/20. 10.1158/0008-5472.CAN-17-0236 . 28522749

[13] Hosoi A, Takeda K, Nagaoka K, Iino T, Matsushita H, Ueha S, et al Increased diversity with reduced "diversity evenness" of tumor infiltrating T-cells for the successful cancer immunotherapy. Scientific reports. 2018;8(1):1058 Epub 2018/01/20. 10.1038/s41598-018-19548-y 29348598 PMC5773695

[14] Dudley ME, Wunderlich JR, Robbins PF, Yang JC, Hwu P, Schwartzentruber DJ, et al Cancer Regression and Autoimmunity in Patients After Clonal Repopulation with Antitumor Lymphocytes. Science. 2002;298(5594):850 10.1126/science.1076514 12242449 PMC1764179

[15] Rosenberg SA, Yang JC, Sherry RM, Kammula US, Hughes MS, Phan GQ, et al Durable complete responses in heavily pretreated patients with metastatic melanoma using T-cell transfer immunotherapy. Clinical cancer research: an official journal of the American Association for Cancer Research. 2011;17(13):4550–7. Epub 2011/04/19. 10.1158/1078-0432.CCR-11-0116 21498393 PMC3131487

[16] Tran E, Turcotte S, Gros A, Robbins PF, Lu YC, Dudley ME, et al Cancer immunotherapy based on mutation-specific CD4+ T cells in a patient with epithelial cancer. Science. 2014;344(6184):641–5. 10.1126/science.1251102 . 24812403 PMC6686185

[17] Zacharakis N, Chinnasamy H, Black M, Xu H, Lu Y-C, Zheng Z, et al Immune recognition of somatic mutations leading to complete durable regression in metastatic breast cancer. Nature medicine. 2018 10.1038/s41591-018-0040-8 29867227 PMC6348479

[18] Stubbington MJT, Rozenblatt-Rosen O, Regev A, Teichmann SA. Single-cell transcriptomics to explore the immune system in health and disease. Science. 2017;358(6359):58 10.1126/science.aan6828 28983043 PMC5654495

[19] Han A, Glanville J, Hansmann L, Davis MM. Linking T-cell receptor sequence to functional phenotype at the single-cell level. Nature biotechnology. 2014;32(7):684–92. Epub 2014/06/24. 10.1038/nbt.2938 24952902 PMC4337815

[20] Heemskerk MH, Hoogeboom M, de Paus RA, Kester MG, van der Hoorn MA, Goulmy E, et al Redirection of antileukemic reactivity of peripheral T lymphocytes using gene transfer of minor histocompatibility antigen HA-2-specific T-cell receptor complexes expressing a conserved alpha joining region. Blood. 2003;102(10):3530–40. Epub 2003/07/19. 10.1182/blood-2003-05-1524 . 12869497

[21] Zhao Y, Zheng Z, Robbins PF, Khong HT, Rosenberg SA, Morgan RA. Primary human lymphocytes transduced with NY-ESO-1 antigen-specific TCR genes recognize and kill diverse human tumor cell lines. Journal of immunology. 2005;174(7):4415–23. Epub 2005/03/22. 10.4049/jimmunol.174.7.4415 15778407 PMC2174604

[22] Rabinovich BA, Ye Y, Etto T, Chen JQ, Levitsky HI, Overwijk WW, et al Visualizing fewer than 10 mouse T cells with an enhanced firefly luciferase in immunocompetent mouse models of cancer. Proceedings of the National Academy of Sciences. 2008;105(38):14342 10.1073/pnas.0804105105 18794521 PMC2567214

[23] Kebriaei P, Singh H, Huls MH, Figliola MJ, Bassett R, Olivares S, et al Phase I trials using Sleeping Beauty to generate CD19-specific CAR T cells. The Journal of clinical investigation. 2016;126(9):3363–76. Epub 2016/08/03. 10.1172/JCI86721 27482888 PMC5004935

[24] Singh H, Huls H, Kebriaei P, Cooper LJ. A new approach to gene therapy using Sleeping Beauty to genetically modify clinical-grade T cells to target CD19. Immunological reviews. 2014;257(1):181–90. Epub 2013/12/18. 10.1111/imr.12137 24329797 PMC4109051

[25] Gibson DG. Enzymatic assembly of overlapping DNA fragments. Methods in enzymology. 2011;498:349–61. Epub 2011/05/24. 10.1016/B978-0-12-385120-8.00015-2 . 21601685 PMC7149801

[26] Lam BY, Zhang W, Ng DC, Maruthappu M, Roderick HL, Chawla S. CREB-dependent Nur77 induction following depolarization in PC12 cells and neurons is modulated by MEF2 transcription factors. J Neurochem. 2010;112(4):1065–73. Epub 2009/12/09. 10.1111/j.1471-4159.2009.06521.x . 19968756

[27] Brickner AG, Warren EH, Caldwell JA, Akatsuka Y, Golovina TN, Zarling AL, et al The immunogenicity of a new human minor histocompatibility antigen results from differential antigen processing. The Journal of experimental medicine. 2001;193(2):195–206. Epub 2001/01/10. 10.1084/jem.193.2.195 11148223 PMC2193344

[28] Hansen TH, Connolly JM, Gould KG, Fremont DH. Basic and translational applications of engineered MHC class I proteins. Trends in immunology. 2010;31(10):363–9. Epub 2010/09/14. 10.1016/j.it.2010.07.003 20832361 PMC2949479

[29] Akatsuka Y, Goldberg TA, Kondo E, Martin EG, Obata Y, Morishima Y, et al Efficient cloning and expression of HLAclass I cDNA in human B-lymphoblastoidcell lines. Tissue Antigens. 2002;59(6):502–11. 10.1034/j.1399-0039.2002.590607.x 12445320

[30] Guo X, Zhang Y, Zheng L, Zheng C, Song J, Zhang Q, et al Global characterization of T cells in non-small-cell lung cancer by single-cell sequencing. Nature medicine. 2018 Epub 2018/06/27. 10.1038/s41591-018-0045-3 . 29942094

[31] Savas P, Virassamy B, Ye C, Salim A, Mintoff CP, Caramia F, et al Single-cell profiling of breast cancer T cells reveals a tissue-resident memory subset associated with improved prognosis. Nature medicine. 2018 Epub 2018/06/27. 10.1038/s41591-018-0078-7 . 29942092

[32] Zemmour D, Zilionis R, Kiner E, Klein AM, Mathis D, Benoist C. Single-cell gene expression reveals a landscape of regulatory T cell phenotypes shaped by the TCR. Nature immunology. 2018 10.1038/s41590-018-0051-0 29434354 PMC6069633

[33] Azizi E, Carr AJ, Plitas G, Cornish AE, Konopacki C, Prabhakaran S, et al Single-Cell Map of Diverse Immune Phenotypes in the Breast Tumor Microenvironment. Cell. 2018 10.1016/j.cell.2018.05.060 29961579 PMC6348010

[34] Hu Z, Anandappa AJ, Sun J, Kim J, Leet DE, Bozym DJ, et al A cloning and expression system to probe T cell receptor specificity and assess functional avidity to neoantigens. Blood. 2018 10.1182/blood-2018-04-843763 30150207 PMC6213317

[35] Pauken KE, Sammons MA, Odorizzi PM, Manne S, Godec J, Khan O, et al Epigenetic stability of exhausted T cells limits durability of reinvigoration by PD-1 blockade. Science. 2016;354(6316):1160–5. Epub 2016/10/30. 10.1126/science.aaf2807 27789795 PMC5484795

[36] Leisegang M, Kammertoens T, Uckert W, Blankenstein T. Targeting human melanoma neoantigens by T cell receptor gene therapy. The Journal of clinical investigation. 2016;126(3):854–8. Epub 2016/01/26. 10.1172/JCI83465 26808500 PMC4767365

[37] Aarnoudse CA, Kruse M, Konopitzky R, Brouwenstijn N, Schrier PI. TCR reconstitution in Jurkat reporter cells facilitates the identification of novel tumor antigens by cDNA expression cloning. Int J Cancer. 2002;99(1):7–13. Epub 2002/04/12. 10.1002/ijc.10317 . 11948485

[38] Petersen TR, Gulland S, Bettelli E, Kuchroo V, Palmer E, Backstrom BT. A chimeric T cell receptor with super-signaling properties. International immunology. 2004;16(7):889–94. Epub 2004/05/19. 10.1093/intimm/dxh098 . 15148288

[39] Hooijberg E, Bakker AQ, Ruizendaal JJ, Spits H. NFAT-controlled expression of GFP permits visualization and isolation of antigen-stimulated primary human T cells. Blood. 2000;96(2):459–66. Epub 2000/07/11. . 10887106

[40] Morimoto S, Fujiki F, Kondo K, Nakajima H, Kobayashi Y, Inatome M, et al Establishment of a novel platform cell line for efficient and precise evaluation of T cell receptor functional avidity. Oncotarget. 2018;9(75):34132–41. Epub 2018/10/23. 10.18632/oncotarget.26139 30344927 PMC6183340

[41] Schaft N, Lankiewicz B, Gratama JW, Bolhuis RLH, Debets R. Flexible and sensitive method to functionally validate tumor-specific receptors via activation of NFAT. Journal of Immunological Methods. 2003;280(1):13–24. 10.1016/S0022-1759(03)00067-X 12972184

[42] Siewert K, Malotka J, Kawakami N, Wekerle H, Hohlfeld R, Dornmair K. Unbiased identification of target antigens of CD8+ T cells with combinatorial libraries coding for short peptides. Nature medicine. 2012;18(5):824–8. Epub 2012/04/10. 10.1038/nm.2720 . 22484809

[43] Barlic J, McDermott DH, Merrell MN, Gonzales J, Via LE, Murphy PM. Interleukin (IL)-15 and IL-2 reciprocally regulate expression of the chemokine receptor CX3CR1 through selective NFAT1- and NFAT2-dependent mechanisms. The Journal of biological chemistry. 2004;279(47):48520–34. Epub 2004/09/07. 10.1074/jbc.M406978200 . 15347678

[44] Diehl S, Chow C-W, Weiss L, Palmetshofer A, Twardzik T, Rounds L, et al Induction of NFATc2 Expression by Interleukin 6 Promotes T Helper Type 2 Differentiation. The Journal of experimental medicine. 2002;196(1):39 10.1084/jem.20020026 12093869 PMC2194007

[45] Ponomarev V, Doubrovin M, Lyddane C, Beresten T, Balatoni J, Bornman W, et al Imaging TCR-dependent NFAT-mediated T-cell activation with positron emission tomography in vivo. Neoplasia (New York, NY). 2001;3(6):480–8. 10.1038/sj/neo/7900204 . 11774030 PMC1506564

[46] Woronicz JD, Lina A, Calnan BJ, Szychowski S, Cheng L, Winoto A. Regulation of the Nur77 orphan steroid receptor in activation-induced apoptosis. Molecular and cellular biology. 1995;15(11):6364 10.1128/mcb.15.11.6364 7565789 PMC230888

[47] Woronicz JD, Calnan B Fau—Ngo V, Ngo V Fau—Winoto A, Winoto A. Requirement for the orphan steroid receptor Nur77 in apoptosis of T-cell hybridomas. 1994;(0028–0836 (Print)). 10.1038/367277a0 8121493

[48] Fassett MS, Jiang W, D'Alise AM, Mathis D, Benoist C. Nuclear receptor Nr4a1 modulates both regulatory T-cell (Treg) differentiation and clonal deletion. Proceedings of the National Academy of Sciences of the United States of America. 2012;109(10):3891–6. Epub 2012/02/22. 10.1073/pnas.1200090109 22345564 PMC3309794

[49] Liu X, Wang Y, Lu H, Li J, Yan X, Xiao M, et al Genome-wide analysis identifies NR4A1 as a key mediator of T cell dysfunction. Nature. 2019 10.1038/s41586-019-0979-8 30814730 PMC6507425

[50] Chen J, López-Moyado IF, Seo H, Lio C-WJ, Hempleman LJ, Sekiya T, et al NR4A transcription factors limit CAR T cell function in solid tumours. Nature. 2019 10.1038/s41586-019-0985-x 30814732 PMC6546093

[51] Guo XZ, Dash P, Calverley M, Tomchuck S, Dallas MH, Thomas PG. Rapid cloning, expression, and functional characterization of paired alphabeta and gammadelta T-cell receptor chains from single-cell analysis. Mol Ther Methods Clin Dev. 2016;3:15054 Epub 2016/02/10. 10.1038/mtm.2015.54 26858965 PMC4729322

[52] Moran AE, Holzapfel KL, Xing Y, Cunningham NR, Maltzman JS, Punt J, et al T cell receptor signal strength in Treg and iNKT cell development demonstrated by a novel fluorescent reporter mouse. The Journal of experimental medicine. 2011;208(6):1279–89. Epub 2011/05/25. 10.1084/jem.20110308 21606508 PMC3173240

[53] Black CB, Duensing TD, Trinkle LS, Dunlay RT. Cell-based screening using high-throughput flow cytometry. Assay Drug Dev Technol. 2011;9(1):13–20. Epub 2010/11/06. 10.1089/adt.2010.0308 21050072 PMC3045571

[54] Colf LA, Bankovich AJ, Hanick NA, Bowerman NA, Jones LL, Kranz DM, et al How a single T cell receptor recognizes both self and foreign MHC. Cell. 2007;129(1):135–46. Epub 2007/04/10. 10.1016/j.cell.2007.01.048 . 17418792

[55] Birnbaum ME, Mendoza JL, Sethi DK, Dong S, Glanville J, Dobbins J, et al Deconstructing the peptide-MHC specificity of T cell recognition. Cell. 2014;157(5):1073–87. Epub 2014/05/27. 10.1016/j.cell.2014.03.047 24855945 PMC4071348

[56] Balakrishnan A, Morris GP. The highly alloreactive nature of dual TCR T cells. Curr Opin Organ Transplant. 2016;21(1):22–8. Epub 2015/11/12. 10.1097/MOT.0000000000000261 26555233 PMC4701647

[57] Hinz T, Weidmann E, Kabelitz D. Dual TCR-expressing T lymphocytes in health and disease. Int Arch Allergy Immunol. 2001;125(1):16–20. Epub 2001/06/01. 10.1159/000053792 . 11385284

[58] Bendle GM, Linnemann C, Hooijkaas AI, Bies L, de Witte MA, Jorritsma A, et al Lethal graft-versus-host disease in mouse models of T cell receptor gene therapy. Nature medicine. 2010;16(5):565–70, 1p following 70. Epub 2010/04/20. 10.1038/nm.2128 . 20400962

[59] Heemskerk MH, Hagedoorn RS, van der Hoorn MA, van der Veken LT, Hoogeboom M, Kester MG, et al Efficiency of T-cell receptor expression in dual-specific T cells is controlled by the intrinsic qualities of the TCR chains within the TCR-CD3 complex. Blood. 2007;109(1):235–43. Epub 2006/09/14. 10.1182/blood-2006-03-013318 . 16968899

[60] Provasi E, Genovese P, Lombardo A, Magnani Z, Liu PQ, Reik A, et al Editing T cell specificity towards leukemia by zinc finger nucleases and lentiviral gene transfer. Nature medicine. 2012;18(5):807–15. Epub 2012/04/03. 10.1038/nm.2700 22466705 PMC5019824

[61] Roth TL, Puig-Saus C, Yu R, Shifrut E, Carnevale J, Li PJ, et al Reprogramming human T cell function and specificity with non-viral genome targeting. Nature. 2018 Epub 2018/07/12. 10.1038/s41586-018-0326-5 . 29995861 PMC6239417

