# Supplementary material for: Very rapid cloning, expression and identifying specificity of T-cell receptors for T-cell engineering
Source: PLoS One. 2020 Feb 10;15(2):e0228112. doi: 10.1371/journal.pone.0228112 (PMC7010234; doi:10.1371/journal.pone.0228112)
Supplement: S1 Fig — (DOCX) [file pone.0228112.s001.docx]

**S1 Fig. Representative sequences of TCR plasmid library**

**Representative coding sequence of a TRAC-2A-TRBV plasmid**

*gat|ATC*CAGAACCCCGACCCCGCCGTGTACCAGCTGAGGGACTCCAAGTCCAGCGACAAGTCCGTGTGCCTGTTCA

<EcoRV>

<-------------------------------TRAC--------------------------------------

CCGACTTCGACTCCCAGACCAACGTGTCCCAGTCCAAGGACTCCGACGTGTACATCACCGACAAGACCGTGCTGGAC

-----------------------------------TRAC--------------------------------------

ATGAGATCCATGGACTTCAAGTCCAACTCCGCCGTGGCCTGGTCCAACAAGTCCGACTTCGCCTGCGCCAACGCCTT

-----------------------------------TRAC--------------------------------------

CAACAACTCCATCATCCCCGAGGACACATTCTTCCCATCCCCCGAGTCCAGCTGCGACGTGAAGCTGGTGGAAAAGT

-----------------------------------TRAC--------------------------------------

CCTTCGAAACCGACACCAACCTGAACTTCCAGAACCTGTCCGTGATCGGCTTCAGGATTCTGCTGCTGAAGGTGGCC

-----------------------------------TRAC--------------------------------------

GGCTTCAACCTGCTGATGACCCTGAGGCTGTGGTCCTCCAGAGCCAAGAGATCCGGCTCCGGCGCCACCAACTTCAG

-------------TRAC---------------------><--Furin---><---SGSGS--><------P2A----

CCTGCTGAAACAGGCCGGCGACGTGGAAGAGAACCCAGGCCCCATGGGCACCCGGCTGTTCTTCTACGTGGCCCTGT

------------------------------------------><-------TRBV----------------------

GTCTGCTGTGGGCCGGACACAGAGATGCCGAGATCACCCAGAGCCCCAGACACAAGATCACCGAGACAGGCAGACAA

-----------------------------------TRBV--------------------------------------

GTGACCCTGGCCTGCCACCAGACCTGGAACCACAACAACATGTTCTGGTACAGACAGGACCTGGGCCACGGCCTGCG

-----------------------------------TRBV--------------------------------------

GCTGATCCACTACTCTTACGGCGTGCAGGACACCAACAAGGGCGAGGTGTCCGACGGCTACAGCGTGTCCAGAAGCA

-----------------------------------TRBV--------------------------------------

ACACCGAGGACCTGCCCCTGACCCTGGAATCTGCCGCCAGCTCTCAGACCAGCGTGTACTTC*TGC|gca*

<FspI>

-----------------------------------TRBV-------------------------->

**Representative coding sequence of a TRAV-TRBC2 plasmid**

ATGTGGGGCGCCTTCCTGCTGTACGTGTCCATGAAGATGGGCGGCACAGCCGGCCAGAGCCTGGAACAGCCTTCTGA

<----------------------------------TRAV--------------------------------------

AGTGACCGCCGTGGAAGGGGCCATCGTGCAGATCAACTGCACCTACCAGACCAGCGGCTTCTACGGCCTGAGCTGGT

-----------------------------------TRAV--------------------------------------

ATCAGCAGCATGACGGCGGAGCCCCCACCTTCCTGAGCTACAATGCCCTGGACGGCCTGGAAGAGACAGGCCGGTTC

-----------------------------------TRAV--------------------------------------

AGCAGCTTCCTGTCCAGAAGCGACAGCTACGGCTACCTGCTGCTGCAGGAACTGCAGATGAAGGACAGCGCCAGCTA

-----------------------------------TRAV--------------------------------------

CTTC*TGC|gca*tttagca*gat|ATC*TGAAGAACGTGTTCCCCCCAGAGGTGGCCGTGTTCGAGCCCTCCGAGGCCGA

<FspI> <EcoRV>

-TRAV-> <-------------TRBC2------------------------------------

GATCTCCCACACCCAGAAAGCCACCCTGGTCTGCCTGGCCACCGGCTTCTACCCTGACCACGTGGAACTGTCTTGGT

-----------------------------------TRBC2-------------------------------------

GGGTGAACGGCAAAGAGGTGCACTCCGGCGTCTCCACCGACCCCCAGCCCCTGAAAGAGCAGCCCGCCCTGAACGAC

-----------------------------------TRBC2-------------------------------------

TCCAGATACTGCCTGTCCTCCAGACTGAGGGTGTCCGCCACCTTCTGGCAGAACCCCAGAAACCACTTCAGGTGCCA

-----------------------------------TRBC2-------------------------------------

GGTGCAGTTCTACGGCCTGTCCGAGAACGACGAGTGGACCCAGGACAGGGCCAAGCCCGTGACACAGATCGTGTCTG

-----------------------------------TRBC2-------------------------------------

CCGAGGCCTGGGGCAGGGCCGACTGCGGATTCACCTCCGAGTCCTACCAGCAGGGCGTGCTGAGCGCCACCATCCTG

-----------------------------------TRBC2-------------------------------------

TACGAGATCCTGCTGGGCAAGGCCACCCTGTACGCCGTGCTGGTGTCCGCTCTGGTGCTGATGGCCATGGTGAAAAG

-----------------------------------TRBC2-------------------------------------

AAAGGACTCCAGGGGCTGA

-----TRBC2-------->
