# Supplementary material for: Very rapid cloning, expression and identifying specificity of T-cell receptors for T-cell engineering
Source: PLoS One. 2020 Feb 10;15(2):e0228112. doi: 10.1371/journal.pone.0228112 (PMC7010234; doi:10.1371/journal.pone.0228112)
Supplement: S2 Fig — (DOCX) [file pone.0228112.s002.docx]

**S2 Fig.**

**Sequences to be added to the 5’ and 3’ ends of CDR3 fragments**

**TCRα CDR3**

3’end of TRA-CDR3 fragment [TRAC]

(N*)ATATCCAGAACCCCGACCCCGCCGTGTACCAGC *Usually, N=A, T

5’end of TRA-CDR3 fragment [TRAV Repertoire specific]

| TRAV | 3’end sequence | Sequence to be added 5’end of CDR3 fragment |
| --- | --- | --- |
| TRAV1_1 | GAACTGCAGATGAAGGACAGCGCCAGCTACTTCTGC  E L Q M K D S A S Y F C | GCAGATGAAGGACAGCGCCAGCTACTTCTGC |
| TRAV1_2 | GAACTGCAGATGAAGGACAGCGCCTCCTACCTGTGC  E L Q M K D S A S Y L C | GCAGATGAAGGACAGCGCCTCCTACCTGTGC |
| TRAV2 | CAAGTGCGCGAAGCCGATGCCGCCGTGTACTACTGC  Q V R E A D A A V Y Y C | GCGCGAAGCCGATGCCGCCGTGTACTACTGC |
| TRAV3 | AGCGCCCTGGTGTCCGACTCCGCCCTGTACTTCTGC  S A L V S D S A L Y F C | CCCTGGTGTCCGACTCCGCCCTGTACTTCTGC |
| TRAV4 | AGAGTGTCCCTGAGCGATACCGCCGTGTACTACTGC  R V S L S D T A V Y Y C | GTCCCTGAGCGATACCGCCGTGTACTACTGC |
| TRAV5 | GATACCCAGACAGGCGACAGCGCCATCTACTTTTGC  D T Q T G D S A I Y F C | CCCAGACAGGCGACAGCGCCATCTACTTTTGC |
| TRAV6 | GCCAGCCAGCCTGCCGATAGCGCCACCTATCTGTGC  A S Q P A D S A T Y L C | GCCAGCCTGCCGATAGCGCCACCTATCTGTGC |
| TRAV7 | GCCGTGCAGCCTGAGGACAGCGCCACCTACTTTTGC  A V Q P E D S A T Y F C | GCAGCCTGAGGACAGCGCCACCTACTTTTGC |
| TRAV8_1 | AGCGTGCAGTGGAGCGATACCGCCGAGTACTTCTGC  S V Q W S D T A E Y F C | GCAGTGGAGCGATACCGCCGAGTACTTCTGC |
| TRAV8_2 | AGCGCCCACATGTCTGATGCCGCCGAGTACTTCTGC  S A H M S D A A E Y F C | CCCACATGTCTGATGCCGCCGAGTACTTCTGC |
| TRAV8_3 | TCCGTGCATTGGAGCGACGCCGCCGAGTACTTCTGC  S V H W S D A A E Y F C | GCATTGGAGCGACGCCGCCGAGTACTTCTGC |
| TRAV8_4 | AGCGCCCACATGTCTGATGCCGCCGAGTACTTCTGC  S A H M S D A A E Y F C | CCCACATGTCTGATGCCGCCGAGTACTTCTGC |
| TRAV8_6_01 | AGCGTGCACATCAGCGATACCGCCGAGTACTTCTGC  S V H I S D T A E Y F C | GCACATCAGCGATACCGCCGAGTACTTCTGC |
| TRAV8_6_02 | AGCGTGCACATCAGCGATACCGCCGAGTACTTCTGC  S V H I S D T A E Y F C | GCACATCAGCGATACCGCCGAGTACTTCTGC |
| TRAV9_1 | AGCGTGCAGGAATCCGACAGCGCCGTGTACTTCTGC  S V Q E S D S A V Y F C | GCAGGAATCCGACAGCGCCGTGTACTTCTGC |
| TRAV9_2 | AGCGTGCAGGTGTCCGACAGCGCCGTGTACTTCTGC  S V Q V S D S A V Y F C | GCAGGTGTCCGACAGCGCCGTGTACTTCTGC |
| TRAV10 | GCCAGCCAGCTGAGCGATAGCGCCAGCTACATCTGC  A S Q L S D S A S Y I C | GCCAGCTGAGCGATAGCGCCAGCTACATCTGC |
| TRAV12 | GACAGCAAGCTGAGCGACAGCGCCACCTACCTGTGC  D S K L S D S A T Y L C | GCAAGCTGAGCGACAGCGCCACCTACCTGTGC |
| TRAV12_2 | GACAGCCAGCCCAGCGACAGCGCCACCTACCTGTGC  D S Q P S D S A T Y L C | GCCAGCCCAGCGACAGCGCCACCTACCTGTGC |
| TRAV12_3 | GACAGCCAGCCCAGCGATTCCGCCACCTATCTGTGC  D S Q P S D S A T Y L C | GCCAGCCCAGCGATTCCGCCACCTATCTGTGC |
| TRAV13_1 | GAGACACAGCCCGAGGACAGCGCCGTGTACTTCTGC  E T Q P E D S A V Y F C | CACAGCCCGAGGACAGCGCCGTGTACTTCTGC |
| TRAV13_2 | GCTACCCAGCCTGGCGATAGCGCCGTGTACTTCTGC  A T Q P G D S A V Y F C | CCCAGCCTGGCGATAGCGCCGTGTACTTCTGC |
| TRAV14_DV4_01 | GCTAGCCAGCTGGGCGACAGCGCCATGTACTTTTGC  A S Q L G D S A M Y F C | GCCAGCTGGGCGACAGCGCCATGTACTTTTGC |
| TRAV14_DV4_02 | GCTAGCCAGCTGGGCGACAGCGCCATGTACTTTTGC  A S Q L G D S A M Y F C | GCCAGCTGGGCGACAGCGCCATGTACTTTTGC |
| TRAV16 | TTCGCCCAGGAAGAGGACAGCGCCATGTACTACTGC  F A Q E E D S A M Y Y C | GCCCAGGAAGAGGACAGCGCCATGTACTACTGC |
| TRAV17 | GCCAGCAGAGCCGCCGATACCGCCAGCTACTTCTGC  A S R A A D T A S Y F C | GCAGAGCCGCCGATACCGCCAGCTACTTCTGC |
| TRAV18 | AGCGTGCAGCTGAGCGACTCCGCCGTGTACTACTGC  S V Q L S D S A V Y Y C | CGTGCAGCTGAGCGACTCCGCCGTGTACTACTGC |
| TRAV19 | GCCAGCCAGGTGGTGGACAGCGCCGTGTACTTCTGC  A S Q V V D S A V Y F C | GCCAGGTGGTGGACAGCGCCGTGTACTTCTGC |
| TRAV20 | GCCCCCAAGCCTGAGGACAGCGCCACCTATCTGTGC  A P K P E D S A T Y L C | CCCAAGCCTGAGGACAGCGCCACCTATCTGTGC |
| TRAV21 | GCTTCTCAGCCTGGCGACTCCGCCACCTATCTGTGC  A S Q P G D S A T Y L C | CTCAGCCTGGCGACTCCGCCACCTATCTGTGC |
| TRAV22 | AGCAGCCAGACCACCGACAGCGGCGTGTACTTCTGC  S S Q T T D S G V Y F C | GCCAGACCACCGACAGCGGCGTGTACTTCTGC |
| TRAV23_DV6 | GACAGCCAGCCCGGCGACAGCGCCACCTACTTTTGC  D S Q P G D S A T Y F C | GCCAGCCCGGCGACAGCGCCACCTACTTTTGC |
| TRAV24 | GGCAGCCAGCCCGAGGACTCCGCCACCTATCTGTGC  G S Q P E D S A T Y L C | GCCAGCCCGAGGACTCCGCCACCTATCTGTGC |
| TRAV25 | GCCACCCAGACCACCGACGTGGGCACCTACTTTTGC  A T Q T T D V G T Y F C | CCCAGACCACCGACGTGGGCACCTACTTTTGC |
| TRAV26_1 | CACGCCACCCTGAGAGATACCGCCGTGTACTACTGC  H A T L R D T A V Y Y C | GCCACCCTGAGAGATACCGCCGTGTACTACTGC |
| TRAV26_2_01 | CGGGCCACCCTGAGAGATGCCGCCGTGTACTACTGC  R A T L R D A A V Y Y C | GCCACCCTGAGAGATGCCGCCGTGTACTACTGC |
| TRAV26_2_02 | GGCCTGACCAGCAACGTGAACAACCGGATGGCCTGC  G L T S N V N N R M A C | CCTGACCAGCAACGTGAACAACCGGATGGCCTGC |
| TRAV27 | GCCGCCCAGCCTGGCGATACCGGCCTGTATCTGTGC  A A Q P G D T G L Y L C | CCCAGCCTGGCGATACCGGCCTGTATCTGTGC |
| TRAV29_DV5 | CCTAGCCAGCCTGGCGATAGCGCCGTGTACTTCTGC  P S Q P G D S A V Y F C | GCCAGCCTGGCGATAGCGCCGTGTACTTCTGC |
| TRAV30 | GCCAGCCAGCTGAGCTACAGCGGCACCTACTTTTGC  A S Q L S Y S G T Y F C | GCCAGCTGAGCTACAGCGGCACCTACTTTTGC |
| TRAV34 | GCCTCCCAGCCTTCTCACGCCGGCATCTACCTGTGC  A S Q P S H A G I Y L C | CCCAGCCTTCTCACGCCGGCATCTACCTGTGC |
| TRAV35 | GCCAGCATCCCCAGCGACGTGGGCATCTACTTTTGC  A S I P S D V G I Y F C | GCATCCCCAGCGACGTGGGCATCTACTTTTGC |
| TRAV36_DV7 | GCCACCCAGACCGGCGACAGCGCCATCTATCTGTGC  A T Q T G D S A I Y L C | CCCAGACCGGCGACAGCGCCATCTATCTGTGC |
| TRAV38_1 | GACAGCCAGCTGGGCGACACCGCCATGTACTTTTGC  D S Q L G D T A M Y F C | GCCAGCTGGGCGACACCGCCATGTACTTTTGC |
| TRAV38_2 | GACAGCCAGCTGGGCGACGCCGCCATGTACTTTTGC  D S Q L G D A A M Y F C | GCCAGCTGGGCGACGCCGCCATGTACTTTTGC |
| TRAV39 | GCCGCCGTGCACGATCTGAGCGCCACCTACTTTTGC  A A V H D L S A T Y F C | CCGTGCACGATCTGAGCGCCACCTACTTTTGC |
| TRAV40 | AGCGTGCAGGTGTCCGACAGCGCCGTGTACTACTGC  S V Q V S D S A V Y Y C | GCAGGTGTCCGACAGCGCCGTGTACTACTGC |
| TRAV41 | GCCAGCCACCCTAGAGACAGCGCCGTGTACATCTGC  A S H P R D S A V Y I C | GCCACCCTAGAGACAGCGCCGTGTACATCTGC |

**TCRβ CDR3**

3’ end of TRB-CDR3 fragment [TRBC2]

GAGG*ATC*TGAAGAACGTGTTCCCCCCAGAGGTGGCC

5’end of TRB-CDR3 fragment [TRBV Repertoire specific]

| TRBV | 3’end sequence | Sequence to be added 5’end of CDR3 fragment |
| --- | --- | --- |
| TRBV2 | AGCACCAAGCTGGAAGATAGCGCCATGTACTTTTGC  S T K L E D S A M Y F C | CCAAGCTGGAAGATAGCGCCATGTACTTTTGC |
| TRBV3_1 | AGCCTGGAACTGGGCGACAGCGCCGTGTACTTCTGC  S L E L G D S A V Y F C | CCTGGAACTGGGCGACAGCGCCGTGTACTTCTGC |
| TRBV4-1 | GCCCTGCAGCCTGAGGACAGCGCCCTGTATCTGTGC  A L Q P E D S A L Y L C | CCTGCAGCCTGAGGACAGCGCCCTGTATCTGTGC |
| TRBV4-2 | ACCCTGCAGCCCGAGGACAGCGCCCTGTATCTGTGC  T L Q P E D S A L Y L C | CCTGCAGCCCGAGGACAGCGCCCTGTATCTGTGC |
| TRBV4-3 | ACCCTGCAGCCCGAGGACAGCGCCCTGTATCTGTGC  T L Q P E D S A L Y L C | CCTGCAGCCCGAGGACAGCGCCCTGTATCTGTGC |
| TRBV5-1 | ACCCTGGAACTGGGCGACAGCGCCCTGTACCTGTGC  T L E L G D S A L Y L C | CCTGGAACTGGGCGACAGCGCCCTGTACCTGTGC |
| TRBV5-4 | GCCCTGGAACTGGACGACAGCGCCCTGTACCTGTGC  A L E L D D S A L Y L C | CCTGGAACTGGACGACAGCGCCCTGTACCTGTGC |
| TRBV5-5 | GCCCTGCTGCTGGGCGATAGCGCCCTGTACCTGTGC  A L L L G D S A L Y L C | CCTGCTGCTGGGCGATAGCGCCCTGTACCTGTGC |
| TRBV5-6 | GCCCTGCTGCTGGGCGATAGCGCCCTGTACCTGTGC  A L L L G D S A L Y L C | CCTGCTGCTGGGCGATAGCGCCCTGTACCTGTGC |
| TRBV5-8 | GCCCTGGAACTGGAAGATAGCGCCCTGTACCTGTGC  A L E L E D S A L Y L C | CCTGGAACTGGAAGATAGCGCCCTGTACCTGTGC |
| TRBV6-1 | AGCGCCGCTCCCAGCCAGACCAGCGTGTACTTCTGC  S A A P S Q T S V Y F C | CCGCTCCCAGCCAGACCAGCGTGTACTTCTGC |
| TRBV6-2 | AGCGCCGCTCCTAGCCAGACCAGCGTGTACTTCTGC  S A A P S Q T S V Y F C | CCGCTCCTAGCCAGACCAGCGTGTACTTCTGC |
| TRBV6-3 | AGCGCCGCTCCTAGCCAGACCAGCGTGTACTTCTGC  S A A P S Q T S V Y F C | CGCTCCTAGCCAGACCAGCGTGTACTTCTGC |
| TRBV6-4_01 | AGCGCCGTGCCTAGCCAGACCAGCGTGTACTTCTGC  S A V P S Q T S V Y F C | CCGTGCCTAGCCAGACCAGCGTGTACTTCTGC |
| TRBV6-4_02 | AGCGCCGTGCCTAGCCAGACCAGCGTGTACTTCTGC  S A V P S Q T S V Y F C | CCGTGCCTAGCCAGACCAGCGTGTACTTCTGC |
| TRBV6-5 | TCTGCCGCCCCATCTCAGACCAGCGTGTACTTCTGC  S A A P S Q T S V Y F C | GCCCCATCTCAGACCAGCGTGTACTTCTGC |
| TRBV6_6_01 | CTGGCTGCCCCATCTCAGACCAGCGTGTACTTCTGC  L A A P S Q T S V Y F C | GCTGCCCCATCTCAGACCAGCGTGTACTTCTGC |
| TRBV6_6_02 | CTGGCTGCCCCATCTCAGACCAGCGTGTACTTCTGC  L A A P S Q T S V Y F C | GCTGCCCCATCTCAGACCAGCGTGTACTTCTGC |
| TRBV6-8 | TCTGCCGCCCCTAGCCAGACCAGCGTGTACCTGTGC  S A A P S Q T S V Y L C | GCCGCCCCTAGCCAGACCAGCGTGTACCTGTGC |
| TRBV6-9 | TCTGCCGCCCCTAGCCAGACCAGCGTGTACTTCTGC  S A A P S Q T S V Y F C | GCCGCCCCTAGCCAGACCAGCGTGTACTTCTGC |
| TRBV7-2_01 | AGAACCCAGCAGGAAGATAGCGCCGTGTACCTGTGC  R T Q Q E D S A V Y L C | CCCAGCAGGAAGATAGCGCCGTGTACCTGTGC |
| TRBV7-2_02 | AGAACCCAGCAGGAAGATAGCGCCGTGTACCTGTGC  R T Q Q E D S A V Y L C | CCCAGCAGGAAGATAGCGCCGTGTACCTGTGC |
| TRBV7-2_03 | AGAACCCAGCAGGAAGATAGCGCCGTGTACCTGTGC  R T Q Q E D S A V Y L C | CCCAGCAGGAAGATAGCGCCGTGTACCTGTGC |
| TRBV7-3_01 | AGAACCGAGCGGGGCGACAGCGCCGTGTATCTGTGT  R T E R G D S A V Y L C | CCGAGCGGGGCGACAGCGCCGTGTATCTGTGT |
| TRBV7-4 | CGGACCGAGCAGGGCGATAGCGCCGTGTATCTGTGC  R T E Q G D S A V Y L C | CCGAGCAGGGCGATAGCGCCGTGTATCTGTGC |
| TRBV7-6 | AGAACCGAGCAGCGGGACAGCGCCATGTACAGATGC  R T E Q R D S A M Y R C | CCGAGCAGCGGGACAGCGCCATGTACAGATGC |
| TRBV7-7 | AGAACCGAGCAGCGGGACAGCGCCATGTACAGATGC  R T E Q R D S A M Y R C | CCGAGCAGCGGGACAGCGCCATGTACAGATGC |
| TRBV7-8_01 | AGAACCCAGCAGGAAGATAGCGCCGTGTACCTGTGC  R T Q Q E D S A V Y L C | CCCAGCAGGAAGATAGCGCCGTGTACCTGTGC |
| TRBV7-8_02 | CGGACCCAGAAAGAGGACAGCGCCGTGTACCTGTGC  R T Q K E D S A V Y L C | CCCAGAAAGAGGACAGCGCCGTGTACCTGTGC |
| TRBV7-9_01 | CGGACCGAGCAGGGCGACAGCGCCATGTATCTGTGC  R T E Q G D S A M Y L C | CCGAGCAGGGCGACAGCGCCATGTATCTGTGC |
| TRBV7-9_03 | CGGACCGAGCAGGGCGACAGCGCCATGTATCTGTGC  R T E Q G D S A M Y L C | CCGAGCAGGGCGACAGCGCCATGTATCTGTGC |
| TRBV9_01 | AGCCTGGAACTGGGCGACAGCGCCCTGTACTTCTGC  S L E L G D S A L Y F C | GGAACTGGGCGACAGCGCCCTGTACTTCTGC |
| TRBV9_02 | AGCCTGGAACTGGGCGACAGCGCCCTGTACTTCTGC  S L E L G D S A L Y F C | GGAACTGGGCGACAGCGCCCTGTACTTCTGC |
| TRBV10_1_01 | TCTGCCGCCAGCTCTCAGACCAGCGTGTACTTCTGC  S A A S S Q T S V Y F C | GCCGCCAGCTCTCAGACCAGCGTGTACTTCTGC |
| TRBV10_1_02 | TCTGCCGCCAGCTCTCAGACCAGCGTGTACTTCTGC  S A A S S Q T S V Y F C | GCCGCCAGCTCTCAGACCAGCGTGTACTTCTGC |
| TRBV10_2 | AGCGCCACCCGGTCCCAGACCAGCGTGTACTTCTGC  S A T R S Q T S V Y F C | GCCACCCGGTCCCAGACCAGCGTGTACTTCTGC |
| TRBV10_3 | AGCGCCACCAGCAGCCAGACCAGCGTGTACTTCTGC  S A T S S Q T S V Y F C | GCCACCAGCAGCCAGACCAGCGTGTACTTCTGC |
| TRBV11_1 | CCTGCCGAGCTGGGCGACAGCGCCATGTATCTGTGC  P A E L G D S A M Y L C | GCCGAGCTGGGCGACAGCGCCATGTATCTGTGC |
| TRBV11_2_01 | CCCGCCAAGCTGGAAGATAGCGCCGTGTACCTGTGC  P A K L E D S A V Y L C | GCCAAGCTGGAAGATAGCGCCGTGTACCTGTGC |
| TRBV11_2_02 | CCCGCCAAGCTGGAAAACAGCGCCGTGTACCTGTGC  P A K L E N S A V Y L C | GCCAAGCTGGAAAACAGCGCCGTGTACCTGTGC |
| TRBV11_3 | CCTGCCGAGCTGGGCGATAGCGCCGTGTATCTGTGC  P A E L G D S A V Y L C | GCCGAGCTGGGCGATAGCGCCGTGTATCTGTGC |
| TRBV12_3 | CCCAGCGAGCCCAGAGACAGCGCCGTGTACTTCTGC  P S E P R D S A V Y F C | GCGAGCCCAGAGACAGCGCCGTGTACTTCTGC |
| TRBV12_4 | CCCAGCGAGCCCAGAGACAGCGCCGTGTACTTCTGC  P S E P R D S A V Y F C | GCGAGCCCAGAGACAGCGCCGTGTACTTCTGC |
| TRBV12_5 | CCCAGCGAGCCCAGAGACAGCGCCGTGTACTTCTGC  P S E P R D S A V Y F C | GCGAGCCCAGAGACAGCGCCGTGTACTTCTGC |
| TRBV13 | AGCCTGGAACTGGGCGACAGCGCCCTGTACTTCTGC  S L E L G D S A L Y F C | CCTGGAACTGGGCGACAGCGCCCTGTACTTCTGC |
| TRBV14 | CCTGCCGAGCTGGAAGATAGCGGCGTGTACTTCTGC  P A E L E D S G V Y F C | GCCGAGCTGGAAGATAGCGGCGTGTACTTCTGC |
| TRBV15 | AGCCCTGGCCTGGGCGACACCGCCATGTACCTGTGC  S P G L G D T A M Y L C | CCTGGCCTGGGCGACACCGCCATGTACCTGTGC |
| TRBV16 | GCCACCAAGCTGGAAGATAGCGCCGTGTACTTCTGC  A T K L E D S A V Y F C | CCAAGCTGGAAGATAGCGCCGTGTACTTCTGC |
| TRBV18 | CAGGTCGTGCGGGGCGATAGCGCCGCCTACTTTTGC  Q V V R G D S A A Y F C | GGTCGTGCGGGGCGATAGCGCCGCCTACTTTTGC |
| TRBV19_01 | AGCGCCCAGAAGAACCCCACCGCCTTCTACCTGTGC  S A Q K N P T A F Y L C | CCCAGAAGAACCCCACCGCCTTCTACCTGTGC |
| TRBV19_02 | AGCGCCCAGAAGAACCCCACCGCCTTCTACCTGTGC  S A Q K N P T A F Y L C | CCCAGAAGAACCCCACCGCCTTCTACCTGTGC |
| TRBV20_1_01 | AGCGCCCACCCCGAGGACAGCAGCTTCTACATCTGC  S A H P E D S S F Y I C | CCCACCCCGAGGACAGCAGCTTCTACATCTGC |
| TRBV20_1_02 | AGCGCCCACCCCGAGGACAGCAGCTTCTACATCTGC  S A H P E D S S F Y I C | CCCACCCCGAGGACAGCAGCTTCTACATCTGC |
| TRBV24_1 | AGCGCCATCCCCAACCAGACCGCCCTGTACTTCTGC  S A I P N Q T A L Y F C | GCCATCCCCAACCAGACCGCCCTGTACTTCTGC |
| TRBV25_1 | AGCGCTAGACCCAGCCACACCAGCCAGTACCTGTGC  S A R P S H T S Q Y L C | GCTAGACCCAGCCACACCAGCCAGTACCTGTGC |
| TRBV27 | AGCCCCAGCCCCAACCAGACCAGCCTGTACTTCTGC  S P S P N Q T S L Y F C | CCCAGCCCCAACCAGACCAGCCTGTACTTCTGC |
| TRBV28 | AGCGCCAGCACCAACCAGACCAGCATGTACCTGTGC  S A S T N Q T S M Y L C | GCCAGCACCAACCAGACCAGCATGTACCTGTGC |
| TRBV29_1 | AACATGAGCCCCGAGGACAGCAGCATCTACCTGTGC  N M S P E D S S I Y L C | CATGAGCCCCGAGGACAGCAGCATCTACCTGTGC |
| TRBV30_02 | AAGCTGCTGCTGAGCGACAGCGGCTTCTACCTGTGC  K L L L S D S G F Y L C | GCTGCTGCTGAGCGACAGCGGCTTCTACCTGTGC |
