# Supplementary material for: Very rapid cloning, expression and identifying specificity of T-cell receptors for T-cell engineering
Source: PLoS One. 2020 Feb 10;15(2):e0228112. doi: 10.1371/journal.pone.0228112 (PMC7010234; doi:10.1371/journal.pone.0228112)
Supplement: S3 Fig — (DOCX) [file pone.0228112.s003.docx]

**S3 Fig.**

**Isolation of antigen-specific T cells and paired CDR3 sequencing of single antigen-specific T cells.**

CD8^+^ T cells isolated from fresh PBMCs and CMV/HLA-A2 pentamer^+^ cells were directly sorted into 96-well plates as single cells. Lysed cells were used to generate cDNA using a TCR constant region specific primer and multiple TCR variable region specific primers (STEP 1). cDNAs were further amplified by multiplex PCR (STEP 2) and subsequent barcoding (STEP 3). Pooled DNA fragments were sequenced using the Illumina MiSeq system (STEP 4).

**
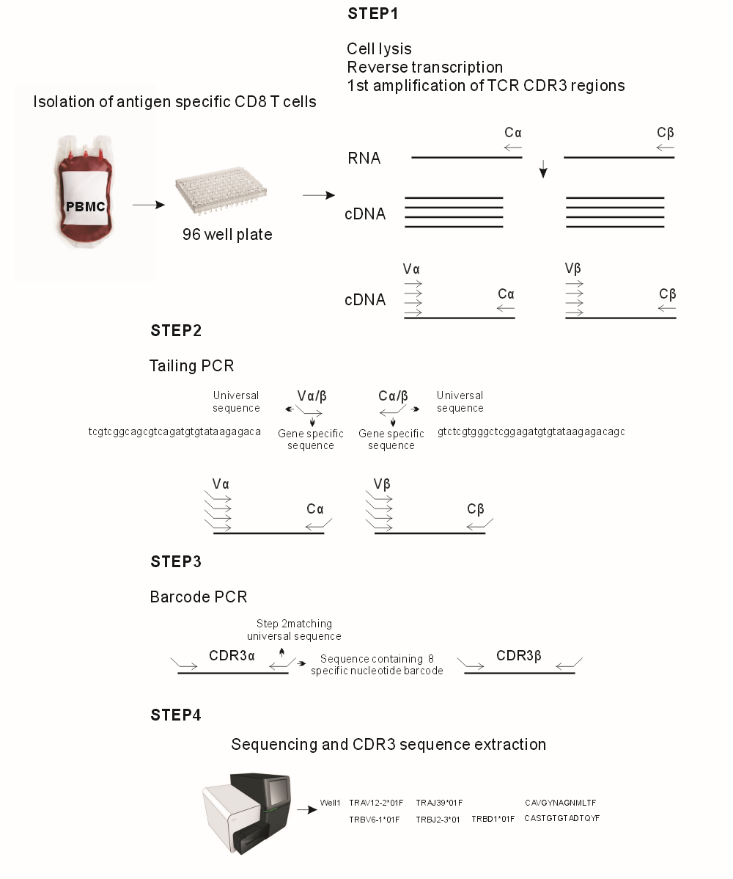
**
