# Supplementary material for: Very rapid cloning, expression and identifying specificity of T-cell receptors for T-cell engineering
Source: PLoS One. 2020 Feb 10;15(2):e0228112. doi: 10.1371/journal.pone.0228112 (PMC7010234; doi:10.1371/journal.pone.0228112)
Supplement: S4 Fig — (DOCX) [file pone.0228112.s004.docx]

**S4 Fig.**

**Sequences for the 2 NR4A1 promoters.**

Sequences highlighted in yellow are myocyte enhancer factor-2 (MEF2) response elements. Sequences highlighted in green are cAMP response element binding protein (CREB) response elements.

**1919 bp promoter (-1800 to +119 from TSS)**

GTCTCAAAGACAACAAAACAAAACAAAAACGGGGAGAGGAAAGGACCCAAGAGATGTTCCAAAAAAGAATTTCAGGAAACAGTGAAAAGTTGCAAATGGGGCATCAGGAGCCAGGAGCAGAGATTAGGTGGCAGGCAGGGGGTGCAGAAATGACAAGTTCTGTCTGGAACAGATGAAATTTGAGGTGCTTGTGGGACCTTTGAGTGGGCAGAAGCGGGGGCTGGAGACAAGGGTGTGGGAAAACTGTGATGGGGGTTGAGGGTGGAAATCGGGCCGGCAGAGCCTCCGGGGCAGAGAAAGTCCTATGGGGTGACCAGATGAGTTGAGAACTGGGTGGGTGGGTGCAGGCTCAATGGAAGCAGAAACTCATGGATATTGACTTACATGGGAGAAGGGGAATGGATGGGGGTCGCAGTGGGGTGGCAGGGCTCTCTTTTCCTTGTTTTTGTTTTTTTGCCCTTCCCTCTTCTCTTACTTTTCCGTGAGGGCTCCCCAGGCTCAGGAGAGATCAGGGTGGAAGGGTGACGGCCAAACCAGGGAAGGCTCCAGGTGGCTGAAGCCTGGTCTGTGCCCAGCAGGGCCCTGGCGGGCTGTTCCTCACTCCACCGGGCAGGTGATAACTGGTCAGAGCTGCCTCCCCACAGGTGCTGGAGGTAGGCTGGGAGGGCCGGTGCTCCCTGATGTGGACAGGGGGAGGGGTATTGATAAGAGGCGTGGAGAGATCCCTAGAGATGCAGTCTGTGGCCCTGGGTTCCAACCCAGTGTGCCACCACCTGGCTGTGTGACCTTCAGCAAGTGCCATTATTTCTCTGAGCCTGTTTGTTTATAAAATGAGGAAGAGTTGGCACAAGTTTGAAAAGATTTCTCAGGCTCCACCCGGTTCTGAAATTCGGTAATTTCCCAACTAGGGTGCACTCCCCCTGTAAGGGGCTGGGGAGGGGACGGTGCGAAACCAAGTTCAGCTTGTGGAGCGGAGCCAGAGCTGTTGGCCGAGCTTGGGCCTGGCCAACGCCTGCCCTCGGGAAGGTCCTGTGTAGGGAGACTGCCTGGAGGGACTAAGCGAGGGCTCTAACTGACGTCTCAGGGGCAGCCTCTCAGCCTGAGACCCTGCTGGGGAAGCCGCGTCCTGTGCACTAGCTGCGCCTAGGGCTGAGGTGAGGGCGCAGGCTCCCCAGGGTGTGTCCGAATTGCCCGCCTCAGCCCGCGGCCTGTCCTGACCGCCCAGCAGCGGCAGCAGCGACACCCTAGGGCTCCAGGAAGGGCTTGGGAAGGTGTAAAGGCGGGGCTAGGCTCGGAGGGAGCCGGAGGGACCGGGCGCGGTTGGCTCCCGGGAGCAACTGGAGAGTGAGGAGATCCTCATCCGGGGAAGCCCCGCGGCCGCGTCTCTACAGCGCCCCTTCTCGGGCTCTGGCCCTCCCGCTGGTTATTCTGGACCTGGGGGCCCCCAGCTGGGACCCGAGTCCGGTGCGGGGAGCCTAGTGGGCCTGGGAGCTGCTATTTTTAGCGGGCGCGGCGGGCGCGAGGAGCCTATTTATAGATCAAACAATCCGCGCTCCCTGCGTCAATGGAACCCCGCGTGCGTCACGCGCGCAGACATTCCAGGCCCCCCCTCCTCGCCCCGCCCCCTCGGGCTCCCCGGGCCGCACCTCCCCCTGGCCGCCTCCCGCCGGAACCGCACCGCCCCCCGCGCCCTTGTATGGCCAAAGCTCGACGGGCGGCCTGCGTCAGTGGCGCCCCCGCCCCTCCCCGTGCGTCACGGAGCGCTTAAGAGGAGGGTCGGGCTCGGCCGGGGAGTCCCAGTGGCGGAGGCTACGAAACTTGGGGGAGTGCACAGAAGAACTTCGGGAGCGCACGCGGGACCAGGGACCAGGCTGAGACTCGGGGCGCCAGTCCGGGCAGGGGCAGCGGGAGCCGGCCGG

**365bp promoter (-319 to +46 from TSS)**

GGCCTGGGAGCTGCTATTTTTAGCGGGCGCGGCGGGCGCGAGGAGCCTATTTATAGATCAAACAATCCGCGCTCCCTGCGTCAATGGAACCCCGCGTGCGTCACGCGCGCAGACATTCCAGGCCCCCCCTCCTCGCCCCGCCCCCTCGGGCTCCCCGGGCCGCACCTCCCCCTGGCCGCCTCCCGCCGGAACCGCACCGCCCCCCGCGCCCTTGTATGGCCAAAGCTCGACGGGCGGCCTGCGTCAGTGGCGCCCCCGCCCCTCCCCGTGCGTCACGGAGCGCTTAAGAGGAGGGTCGGGCTCGGCCGGGGAGTCCCAGTGGCGGAGGCTACGAAACTTGGGGGAGTGCACAGAAGAACTTCGGG
