# Supplementary material for: Very rapid cloning, expression and identifying specificity of T-cell receptors for T-cell engineering
Source: PLoS One. 2020 Feb 10;15(2):e0228112. doi: 10.1371/journal.pone.0228112 (PMC7010234; doi:10.1371/journal.pone.0228112)

**S5 Fig**

**Fluorescence microscopy images of JRFTCRs stimulated with cognate antigen.**

JFTCRs transfected with NY-ESO-1/HLA-A2−specific TCRs were stimulated with cognate peptide−pulsed irrelevant peptide−pulsed HLA-A2 transduced 721.221 cells

Images were captured 48 hours after stimulatioin using an EVOS-FL Cell Imaging System (Thermo Fisher Scientific). Unstimulated and OKT3-stimulated JFTCRs transfected with NY-ESO-1/HLA-A2−specific TCR were the negative and positive controls, respectively.


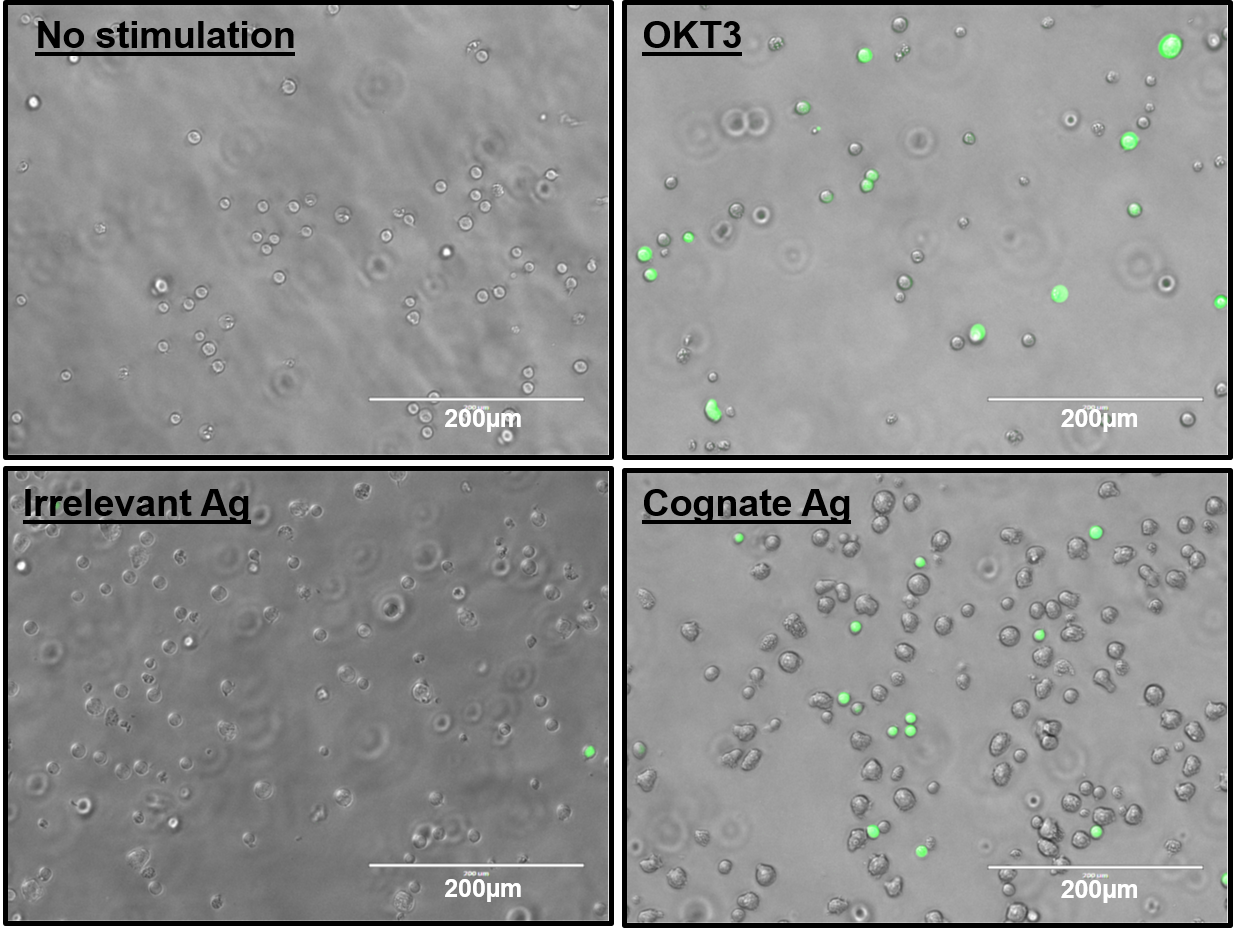

Supplement: S5 Fig — (DOCX) [file pone.0228112.s005.docx]
