# Supplementary material for: Very rapid cloning, expression and identifying specificity of T-cell receptors for T-cell engineering
Source: PLoS One. 2020 Feb 10;15(2):e0228112. doi: 10.1371/journal.pone.0228112 (PMC7010234; doi:10.1371/journal.pone.0228112)
Supplement: S6 Fig — (DOCX) [file pone.0228112.s006.docx]

**S6 Fig.**

**Reporter assay comparing JRFTCRs transduced with high- or low-affinity NY-ESO-1/HLA-A2−specific TCRαβs.**

**a.**

**
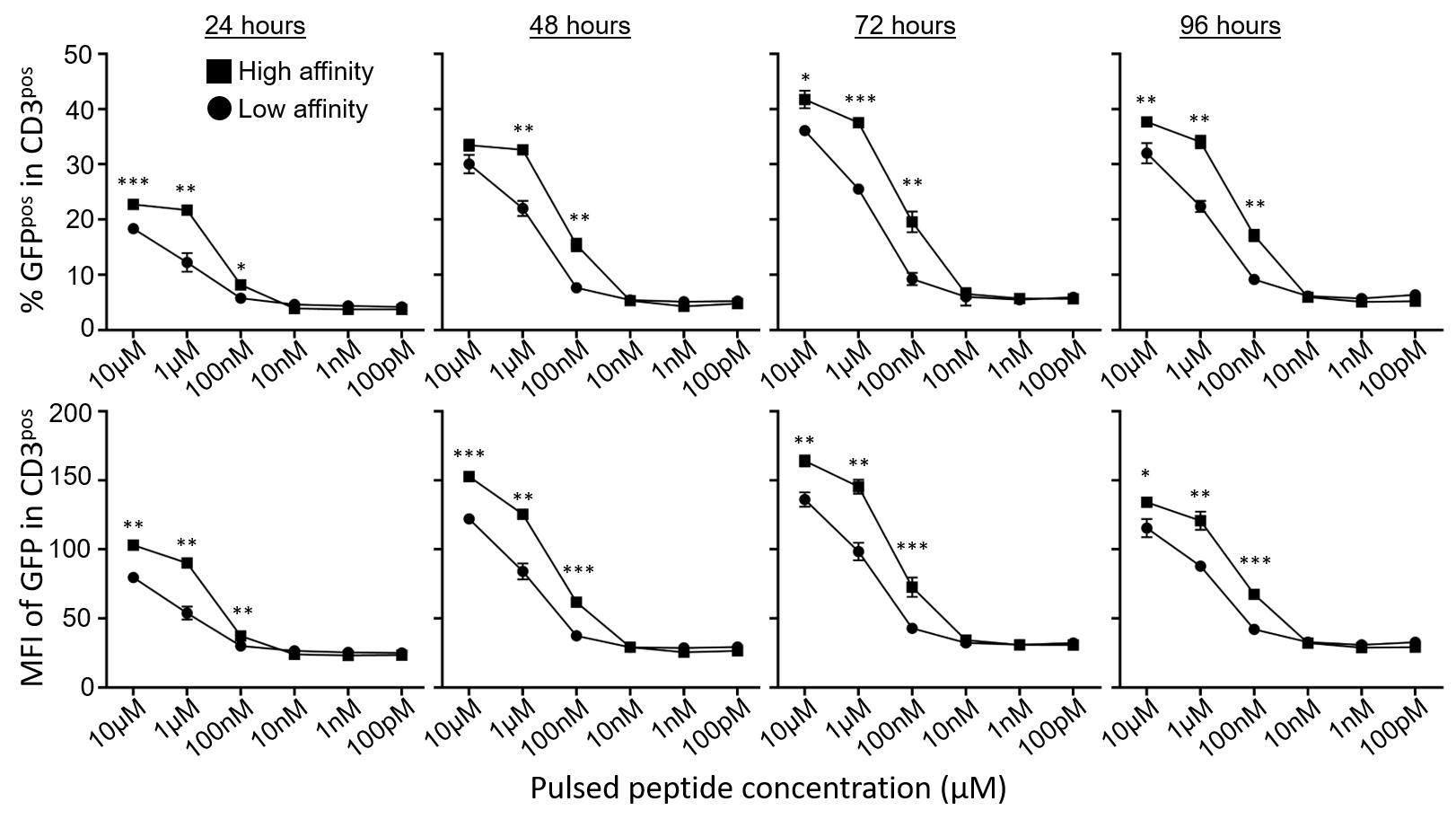
**

**b.**

**
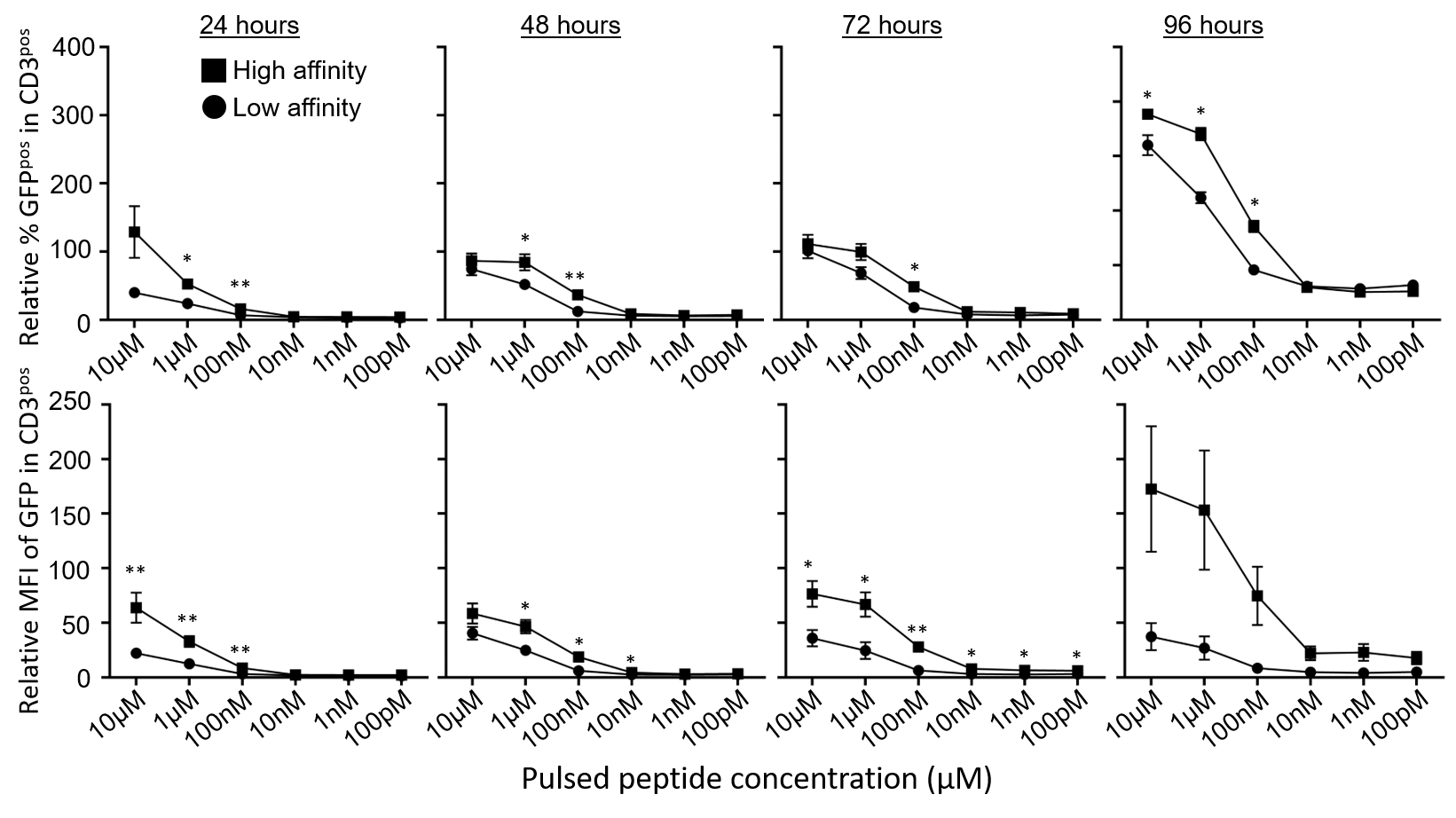
**
