# Supplementary material for: Very rapid cloning, expression and identifying specificity of T-cell receptors for T-cell engineering
Source: PLoS One. 2020 Feb 10;15(2):e0228112. doi: 10.1371/journal.pone.0228112 (PMC7010234; doi:10.1371/journal.pone.0228112)
Supplement: S7 Fig — (DOCX) [file pone.0228112.s007.docx]

**S7 Fig.**

**CD3 expression of TCR-transfected JRFTCRs after antigen-specific stimulation.**

After antigen-specific stimulation, TCR-transfected JRFTCRs were monitored for CD3 expression. After gating in the CD8^+^ population (JRFTCRs), CD3 expression was measured as the ratio of MFI to CD3 expression on unstimulated TCR-transfected JRFTCRs.

Data were obtained from JRFTCRs transfected with NY-ESO-1/HLA-A2−specific TCR and stimulated with NY-ESO-1/A2 peptide−pulsed HLA-A2−transduced 721.221 cells.

***p < 0.001; N.S., not significant.
