# Supplementary material for: Very rapid cloning, expression and identifying specificity of T-cell receptors for T-cell engineering
Source: PLoS One. 2020 Feb 10;15(2):e0228112. doi: 10.1371/journal.pone.0228112 (PMC7010234; doi:10.1371/journal.pone.0228112)
Supplement: S8 Fig — (DOCX) [file pone.0228112.s008.docx]

**S8 Fig.**

**
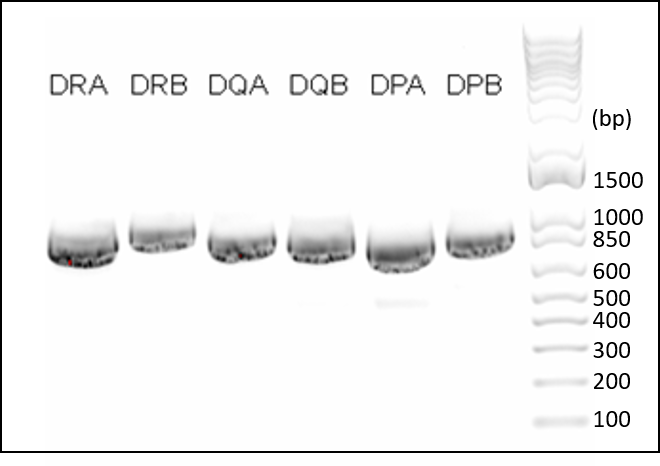
**

**PCR cloning of HLA class II using a universal primer pair for each HLA class II locus.**

Representative agarose gel image of amplified HLA class II from PBMC. The primers used to amplify each HLA class II locus are shown below.

Lower case letters indicate overlap sequences for Gibson assembly. gccacc: Kozak sequence.

HLA-DRA forward:

cgcagtcagtgctctagagctagcggccaccATGGCCATAAGTGGAGTCCCTGTGCTAGG

HLA-DRA reverse:

gtaatccagaggttgattgtcgacgcTTACAGAGGCCCCCTGCGTTCTGCTGC

HLA-DRB forward:

gcagtcagtgctctagagctagcggccaccATGGTGTGTCTGARGYTCCCTGGAKGYTCC

HLA-DRB reverse:

gtaatccagaggttgattgtcgacgcTCAGCTCAVGARTCCTSTTGGSKGRAGTCC

HLA-DQA forward:

gcagtcagtgctctagagctagcggccaccATGATCCTAAACAAAGCTCTGMTGCTGGGG

HLA-DQA reverse:

gtaatccagaggttgattgtcgacgcTCACAAKGGCCCYTGGTGTCTGGAAGCACC

HLA-DQB forward:

cgcagtcagtgctctagagctagcggccaccATGTCTTGGAARAAGKCTTTGCGGATCCCYGGAG

HLA-DQB reverse:

gtaatccagaggttgattgtcgacgcTCAGTGCAGRAGCCCTTTCYGACTCCTKTGAYGG

HLA-DPA forward:

cgcagtcagtgctctagagctagcggccaccATGCGCCCWGAAGACAGAATGTTCCATATCAGAGC

HLA-DPA reverse:

gtaatccagaggttgattgtcgacgcTCACAGGGKCCCCTGGGCCCGGGG

HLA-DPB forward:

cgcagtcagtgctctagagctagcggccaccATGATGGTTCTGCAGGTTTCTGCRGCCCC

HLA-DPB reverse:

gtaatccagaggttgattgtcgacgcTTATGCAGATCCTCGYTGAACTTTCTTGCTCCTCCTGTGC
