# Supplementary material for: Very rapid cloning, expression and identifying specificity of T-cell receptors for T-cell engineering
Source: PLoS One. 2020 Feb 10;15(2):e0228112. doi: 10.1371/journal.pone.0228112 (PMC7010234; doi:10.1371/journal.pone.0228112)
Supplement: S1 Table — (DOCX) [file pone.0228112.s009.docx]

**S1 Table.**

**Primers for the initial amplification of the CDR3 regions of TCRα and TCRβ.**

| **Name** | **Sequence** |
| --- | --- |
| TRAV-1 | CTGCACGTACCAGACATCTGGGTT |
| TRAV2 | GGCTCAAAGCCTTCTCAGCAGG |
| TRAV3 | GGATAACCTGGTTAAAGGCAGCTA |
| TRAV4 | GGATACAAGACAAAAGTTACAAACGA |
| TRAV5 | GCTGACGTATATTTTTTCAAATATGGA |
| TRAV6 | GGAAGAGGCCCTGTTTTCTTGCT |
| TRAV7 | GCTGGATATGAGAAGCAGAAAGGA |
| TRAV8 | AGGACTCCAGCTTCTCCTGAAGTA |
| TRAV9 | GTATGTCCAATATCCTGGAGAAGGT |
| TRAV10 | CAGTGAGAACACAAAGTCGAACGG |
| TRAV12.1 | CCTAAGTTGCTGATGTCCGTATAC |
| TRAV12.2 | GGGAAAAGCCCTGAGTTGATAATGT |
| TRAV12.3 | GCTGATGTACACATACTCCAGTGG |
| TRAV13.1 | CCCTTGGTATAAGCAAGAACTTGG |
| TRAV13.2 | CCTCAATTCATTATAGACATTCGTTC |
| TRAV14 | GCAAAATGCAACAGAAGGTCGCTA |
| TRAV16 | TAGAGAGAGCATCAAAGGCTTCAC |
| TRAV17 | CGTTCAAATGAAAGAGAGAAACACAG |
| TRAV18 | CCTGAAAAGTTCAGAAAACCAGGAG |
| TRAV19 | GGTCGGTATTCTTGGAACTTCCAG |
| TRAV20 | GCTGGGGAAGAAAAGGAGAAAGAAA |
| TRAV21 | GTCAGAGAGAGCAAACAAGTGGAA |
| TRAV22 | GGACAAAACAGAATGGAAGATTAAGC |
| TRAV23 | CCAGATGTGAGTGAAAAGAAAGAAG |
| TRAV24 | GACTTTAAATGGGGATGAAAAGAAGA |
| TRAV25 | GGAGAAGTGAAGAAGCAGAAAAGAC |
| TRAV26.1 | CCAATGAAATGGCCTCTCTGATCA |
| TRAV26.2 | GCAATGTGAACAACAGAATGGCCT |
| TRAV27 | GGTGGAGAAGTGAAGAAGCTGAAG |
| TRAV29 | GGATAAAAATGAAGATGGAAGATTCAC |
| TRAV30 | CCTGATGATATTACTGAAGGGTGGA |
| TRAV34 | GGTGGGGAAGAGAAAAGTCATGAA |
| TRAV35 | GGTGAATTGACCTCAAATGGAAGAC |
| TRAV36 | GCTAACTTCAAGTGGAATTGAAAAGA |
| TRAV38 | GAAGCTTATAAGCAACAGAATGCAAC |
| TRAV39 | GGAGCAGTGAAGCAGGAGGGAC |
| TRAV40 | GAGAGACAATGGAAAACAGCAAAAAC |
| TRAV41 | GCTGAGCTCAGGGAAGAAGAAGC |
| TRBV2 | CTGAAATATTCGATGATCAATTCTCAG |
| TRBV3-1 | TCATTATAAATGAAACAGTTCCAAATCG |
| TRBV4 | AGTGTGCCAAGTCGCTTCTCAC |
| TRBV5-4,8 | CAGAGGAAACTYCCCTCCTAGATT |
| TRBV5-1 | GAGACACAGAGAAACAAAGGAAACTTC |
| **Name** | **Sequence** |
| TRBV6-1 | GGTACCACTGACAAAGGAGAAGTCC |
| TRBV6-2,3 | GAGGGTACAACTGCCAAAGGAGAGGT |
| TRBV6-4 | GGCAAAGGAGAAGTCCCTGATGGTT |
| TRBV6-5,6 | AAGGAGAAGTCCCSAATGGCTACAA |
| TRBV6-8 | CTGACAAAGAAGTCCCCAATGGCTAC |
| TRBV6-9 | CACTGACAAAGGAGAAGTCCCCGAT |
| TRBV7-2 | AGACAAATCAGGGCTGCCCAGTGA |
| TRBV7-3 | GACTCAGGGCTGCCCAACGAT |
| TRBV7-8 | CCAGAATGAAGCTCAACTAGACAA |
| TRBV7-4,6 | GGTTCTCTGCAGAGAGGCCTGAG |
| TRBV7-1 | GGCTGCCCAGTGATCGGTTCTC |
| TRBV7-9 | GACTTACTTCCAGAATGAAGCTCAACT |
| TRBV-9 | GAGCAAAAGGAAACATTCTTGAACGATT |
| TRBV10-1,3 | GGCTRATCCATTACTCATATGGTGTT |
| TRBV10-2 | GATAAAGGAGAAGTCCCCGATGGCT |
| TRBV11 | GATTCACAGTTGCCTAAGGATCGAT |
| TRBV12-3,4 | GATTCAGGGATGCCCGAGGATCG |
| TRBV12-5 | GATTCGGGGATGCCGAAGGATCG |
| TRBV13 | GCAGAGCGATAAAGGAAGCATCCCT |
| TRBV14 | TCCGGTATGCCCAACAATCGATTCT |
| TRBV15 | GATTTTAACAATGAAGCAGACACCCCT |
| TRBV16 | GATGAAACAGGTATGCCCAAGGAAAG |
| TRBV18 | TATCATAGATGAGTCAGGAATGCCAAAG |
| TRBV19 | GACTTTCAGAAAGGAGATATAGCTGAA |
| TRBV20-1 | CAAGGCCACATACGAGCAAGGCGTC |
| TRBV24-1 | CAAAGATATAAACAAAGGAGAGATCTCT |
| TRBV25-1 | AGAGAAGGGAGATCTTTCCTCTGAGT |
| TRBV27-1 | GACTGATAAGGGAGATGTTCCTGAAG |
| TRBV28 | GGCTGATCTATTTCTCATATGATGTTAA |
| TRBV29 | GCCACATATGAGAGTGGATTTGTCATT |
| TRBV30 | GGTGCCCCAGAATCTCTCAGCCT |
| TRAC | CGGTGAATAGGCAGACAGACTTGT |
| TRBC | ACCAGTGTGGCCTTTTGGGTGTG |
