# Supplementary material for: Very rapid cloning, expression and identifying specificity of T-cell receptors for T-cell engineering
Source: PLoS One. 2020 Feb 10;15(2):e0228112. doi: 10.1371/journal.pone.0228112 (PMC7010234; doi:10.1371/journal.pone.0228112)
Supplement: S2 Table — (DOCX) [file pone.0228112.s010.docx]

**S2 Table.**

**Barcoded primers for the CDR3 regions of TCRα and TCRβ.**

| **Name** | **Sequence** |
| --- | --- |
| TRAV-1 | tcgtcggcagcgtcagatgtgtataagagacaAGGTCGTTTTTCTTCATTCCTTAGTC |
| TRAV2 | tcgtcggcagcgtcagatgtgtataagagacaACGATACAACATGACCTATGAACGG |
| TRAV3 | tcgtcggcagcgtcagatgtgtataagagacaCTTTGAAGCTGAATTTAACAAGAGCC |
| TRAV4.1 | tcgtcggcagcgtcagatgtgtataagagacaCTCCCTGTTTATCCCTGCCGAC |
| TRAV5.1 | tcgtcggcagcgtcagatgtgtataagagacaAAACAACACCAAAGACTCACTGTTC |
| TRAV6 | tcgtcggcagcgtcagatgtgtataagagacaAAGACTGAAGGTCACCTTTGATACC |
| TRAV7 | tcgtcggcagcgtcagatgtgtataagagacaACTAAATGCTACATTACTGAAGAATGG |
| TRAV8 | tcgtcggcagcgtcagatgtgtataagagacaGCATCAACGGTTTTCAGGCTGAATTTAA |
| TRAV9 | tcgtcggcagcgtcagatgtgtataagagacaGAAACCACTTCTTTCCACTTGGAGAA |
| TRAV10 | tcgtcggcagcgtcagatgtgtataagagacaTACAGCAACTCTGGATGCAGACAC |
| TRAV12 | tcgtcggcagcgtcagatgtgtataagagacaGAAGATGGAAGGTTTACAGCACA |
| TRAV13.1 | tcgtcggcagcgtcagatgtgtataagagacaGACATTGGTTCAAATGTGGGCGAA |
| TRAV13.2 | tcgtcggcagcgtcagatgtgtataagagacaGGCAAGGCCAAAGAGTCACCGT |
| TRAV14 | tcgtcggcagcgtcagatgtgtataagagacaTCCAGAAGGCAAGAAAATCCGCCA |
| TRAV16 | tcgtcggcagcgtcagatgtgtataagagacaGCTGACCTTAACAAAGGCGAGACA |
| TRAV17 | tcgtcggcagcgtcagatgtgtataagagacaTTAAGACTCACGCTTGACACTTCCA |
| TRAV18 | tcgtcggcagcgtcagatgtgtataagagacaGCAGAGCTTTTCAGGCCAGTCCT |
| TRAV19 | tcgtcggcagcgtcagatgtgtataagagacaTCCACCAGTTCCTTCAACTTCACC |
| TRAV20 | tcgtcggcagcgtcagatgtgtataagagacaGCCACATTAACAAAGAAGGAAAGCT |
| TRAV21 | tcgtcggcagcgtcagatgtgtataagagacaGCCTCGCTGGATAAATCATCAGGA |
| TRAV22 | tcgtcggcagcgtcagatgtgtataagagacaACGACTGTCGCTACGGAACGCTA |
| TRAV23 | tcgtcggcagcgtcagatgtgtataagagacaCACAATCTCCTTCAATAAAAGTGCCA |
| TRAV24 | tcgtcggcagcgtcagatgtgtataagagacaACGAATAAGTGCCACTCTTAATACCA |
| TRAV25 | tcgtcggcagcgtcagatgtgtataagagacaGTTTGGAGAAGCAAAAAAGAACAGCT |
| TRAV26.1 | tcgtcggcagcgtcagatgtgtataagagacaCAGAAGACAGAAAGTCCAGCACCT |
| TRAV26.2 | tcgtcggcagcgtcagatgtgtataagagacaATCGCTGAAGACAGAAAGTCCAGT |
| TRAV27 | tcgtcggcagcgtcagatgtgtataagagacaACTAACCTTTCAGTTTGGTGATGCAA |
| TRAV29 | tcgtcggcagcgtcagatgtgtataagagacaCTTAAACAAAAGTGCCAAGCACCTC |
| TRAV30 | tcgtcggcagcgtcagatgtgtataagagacaAATATCTGCTTCATTTAATGAAAAAAAGC |
| TRAV34 | tcgtcggcagcgtcagatgtgtataagagacaCCAAGTTGGATGAGAAAAAGCAGCA |
| TRAV35 | tcgtcggcagcgtcagatgtgtataagagacaCTCAGTTTGGTATAACCAGAAAGGA |
| TRAV36 | tcgtcggcagcgtcagatgtgtataagagacaGGAAGACTAAGTAGCATATTAGATAAG |
| TRAV38 | tcgtcggcagcgtcagatgtgtataagagacaCTGTGAACTTCCAGAAAGCAGCCA |
| TRAV39 | tcgtcggcagcgtcagatgtgtataagagacaCCTCACTTGATACCAAAGCCCGT |
| TRAV40 | tcgtcggcagcgtcagatgtgtataagagacaAGGCGGAAATATTAAAGACAAAAACTC |
| TRAV41 | tcgtcggcagcgtcagatgtgtataagagacaGATTAATTGCCACAATAAACATACAGG |
| TRBV2 | tcgtcggcagcgtcagatgtgtataagagacaGCCTGATGGATCAAATTTCACTCTG |
| TRBV3-1 | tcgtcggcagcgtcagatgtgtataagagacaTCTCACCTAAATCTCCAGACAAAGCT |
| TRBV4 | tcgtcggcagcgtcagatgtgtataagagacaCCTGAATGCCCCAACAGCTCTC |
| TRBV5-4,8 | tcgtcggcagcgtcagatgtgtataagagacaCTCTGAGCTGAATGTGAACGCCT |
| TRBV5-1 | tcgtcggcagcgtcagatgtgtataagagacaCGATTCTCAGGGCGCCAGTTCTCT |
| TRBV6-1 | tcgtcggcagcgtcagatgtgtataagagacaTGGCTACAATGTCTCCAGATTAAACAA |
| TRBV6-2,3 | tcgtcggcagcgtcagatgtgtataagagacaCCCTGATGGCTACAATGTCTCCAGA |
| **Name** | **Sequence** |
| TRBV6-4 | tcgtcggcagcgtcagatgtgtataagagacaGTGTCTCCAGAGCAAACACAGATGATT |
| TRBV6-5,6 | tcgtcggcagcgtcagatgtgtataagagacaGTCTCCAGATCAACCACAGAGGAT |
| TRBV6-8 | tcgtcggcagcgtcagatgtgtataagagacaGTCTCTAGATTAAACACAGAGGATTTC |
| TRBV6-9 | tcgtcggcagcgtcagatgtgtataagagacaGGCTACAATGTATCCAGATCAAACA |
| TRBV7-2 | tcgtcggcagcgtcagatgtgtataagagacaTCGCTTCTCTGCAGAGAGGACTGG |
| TRBV7-3 | tcgtcggcagcgtcagatgtgtataagagacaCGGTTCTTTGCAGTCAGGCCTGA |
| TRBV7-8 | tcgtcggcagcgtcagatgtgtataagagacaCCAGTGATCGCTTCTTTGCAGAAA |
| TRBV7-4,6 | tcgtcggcagcgtcagatgtgtataagagacaTCTCCACTCTGAMGATCCAGCGCA |
| TRBV7-7 | tcgtcggcagcgtcagatgtgtataagagacaGCAGAGAGGCCTGAGGGATCCAT |
| TRBV7-9 | tcgtcggcagcgtcagatgtgtataagagacaCTGCAGAGAGGCCTAAGGGATCT |
| TRBV-9 | tcgtcggcagcgtcagatgtgtataagagacaCTCCGCACAACAGTTCCCTGACTT |
| TRBV10-1,3 | tcgtcggcagcgtcagatgtgtataagagacaCAGATGGCTAYAGTGTCTCTAGATCAAA |
| TRBV10-2 | tcgtcggcagcgtcagatgtgtataagagacaGTTGTCTCCAGATCCAAGACAGAGAA |
| TRBV11 | tcgtcggcagcgtcagatgtgtataagagacaGCAGAGAGGCTCAAAGGAGTAGACT |
| TRBV12-3,4 | tcgtcggcagcgtcagatgtgtataagagacaGCTAAGATGCCTAATGCATCATTCTC |
| TRBV12-5 | tcgtcggcagcgtcagatgtgtataagagacaCTCAGCAGAGATGCCTGATGCAACT |
| TRBV13 | tcgtcggcagcgtcagatgtgtataagagacaTCTCAGCTCAACAGTTCAGTGACTA |
| TRBV14 | tcgtcggcagcgtcagatgtgtataagagacaGCTGAAAGGACTGGAGGGACGTAT |
| TRBV15 | tcgtcggcagcgtcagatgtgtataagagacaGATAACTTCCAATCCAGGAGGCCG |
| TRBV16 | tcgtcggcagcgtcagatgtgtataagagacaGCTAAGTGCCTCCCAAATTCACCC |
| TRBV18 | tcgtcggcagcgtcagatgtgtataagagacaGGAACGATTTTCTGCTGAATTTCCCA |
| TRBV19 | tcgtcggcagcgtcagatgtgtataagagacaGGTACAGCGTCTCTCGGGAGAAGA |
| TRBV20-1 | tcgtcggcagcgtcagatgtgtataagagacaGGACAAGTTTCTCATCAACCATGCAA |
| TRBV24-1 | tcgtcggcagcgtcagatgtgtataagagacaTGGATACAGTGTCTCTCGACAGGC |
| TRBV25-1 | tcgtcggcagcgtcagatgtgtataagagacaCAACAGTCTCCAGAATAAGGACGGA |
| TRBV27-1 | tcgtcggcagcgtcagatgtgtataagagacaTACAAAGTCTCTCGAAAAGAGAAGAGGA |
| TRBV28 | tcgtcggcagcgtcagatgtgtataagagacaGGGGTACAGTGTCTCTAGAGAGA |
| TRBV29 | tcgtcggcagcgtcagatgtgtataagagacaGTTTCCCATCAGCCGCCCAAACCTA |
| TRBV30 | tcgtcggcagcgtcagatgtgtataagagacaCAGACCCCAGGACCGGCAGTTCAT |
| TRAC | gtctcgtgggctcggagatgtgtataagagacagcAGACAGACTTGTCACTGGATTTAG |
| TRBC | gtctcgtgggctcggagatgtgtataagagacagcTTTTGGGTGTGGGAGATCTCTG |
