# Supplementary material for: Very rapid cloning, expression and identifying specificity of T-cell receptors for T-cell engineering
Source: PLoS One. 2020 Feb 10;15(2):e0228112. doi: 10.1371/journal.pone.0228112 (PMC7010234; doi:10.1371/journal.pone.0228112)
Supplement: S3 Table — (DOCX) [file pone.0228112.s011.docx]

**S3 Table.**

**5’ and 3’ barcoding primers for NGS of the CDR3 regions of TCRα and TCRβ.**

5’-Barcoding primers

| **Name** | **Sequence** |
| --- | --- |
| I501 | AATGATACGGCGACCACCGAGATCTACAC**TAGATCGC**TCGTCGGCAGCGTC |
| I502 | AATGATACGGCGACCACCGAGATCTACAC**CTCTCTAT**TCGTCGGCAGCGTC |
| I503 | AATGATACGGCGACCACCGAGATCTACAC**TATCCTCT**TCGTCGGCAGCGTC |
| I504 | AATGATACGGCGACCACCGAGATCTACAC**AGAGTAGA**TCGTCGGCAGCGTC |
| I505 | AATGATACGGCGACCACCGAGATCTACAC**GTAAGGAG**TCGTCGGCAGCGTC |
| I506 | AATGATACGGCGACCACCGAGATCTACAC**ACTGCATA**TCGTCGGCAGCGTC |
| I507 | AATGATACGGCGACCACCGAGATCTACAC**AAGGAGTA**TCGTCGGCAGCGTC |
| I508 | AATGATACGGCGACCACCGAGATCTACAC**CTAAGCCT**TCGTCGGCAGCGTC |
| I509 | AATGATACGGCGACCACCGAGATCTACAC**CGTCTAAT**TCGTCGGCAGCGTC |
| I510 | AATGATACGGCGACCACCGAGATCTACAC**TCTCTCCG**TCGTCGGCAGCGTC |
| I511 | AATGATACGGCGACCACCGAGATCTACAC**TCGACTAG**TCGTCGGCAGCGTC |
| I512 | AATGATACGGCGACCACCGAGATCTACAC**TTCTAGCT**TCGTCGGCAGCGTC |

3’-Barcoding primers

| **Name** | **Sequence** |
| --- | --- |
| I701 | CAAGCAGAAGACGGCATACGAGAT**TCGCCTTA**GTCTCGTGGGCTCGG |
| I702 | CAAGCAGAAGACGGCATACGAGAT**CTAGTACG**GTCTCGTGGGCTCGG |
| I703 | CAAGCAGAAGACGGCATACGAGAT**TTCTGCCT**GTCTCGTGGGCTCGG |
| I704 | CAAGCAGAAGACGGCATACGAGAT**GCTCAGGA**GTCTCGTGGGCTCGG |
| I705 | CAAGCAGAAGACGGCATACGAGAT**AGGAGTCC**GTCTCGTGGGCTCGG |
| I706 | CAAGCAGAAGACGGCATACGAGAT**CATGCCTA**GTCTCGTGGGCTCGG |
| I707 | CAAGCAGAAGACGGCATACGAGAT**GTAGAGAG**GTCTCGTGGGCTCGG |
| I708 | CAAGCAGAAGACGGCATACGAGAT**CCTCTCTG**GTCTCGTGGGCTCGG |

5’- and 3’ barcoding primers combination in 96 well plate
