# Supplementary figures and images for: Very rapid cloning, expression and identifying specificity of T-cell receptors for T-cell engineering
Source: PLoS One. 2020 Feb 10;15(2):e0228112. doi: 10.1371/journal.pone.0228112 (PMC7010234; doi:10.1371/journal.pone.0228112)

PCRs for HLA cloning from Melanoma DTS

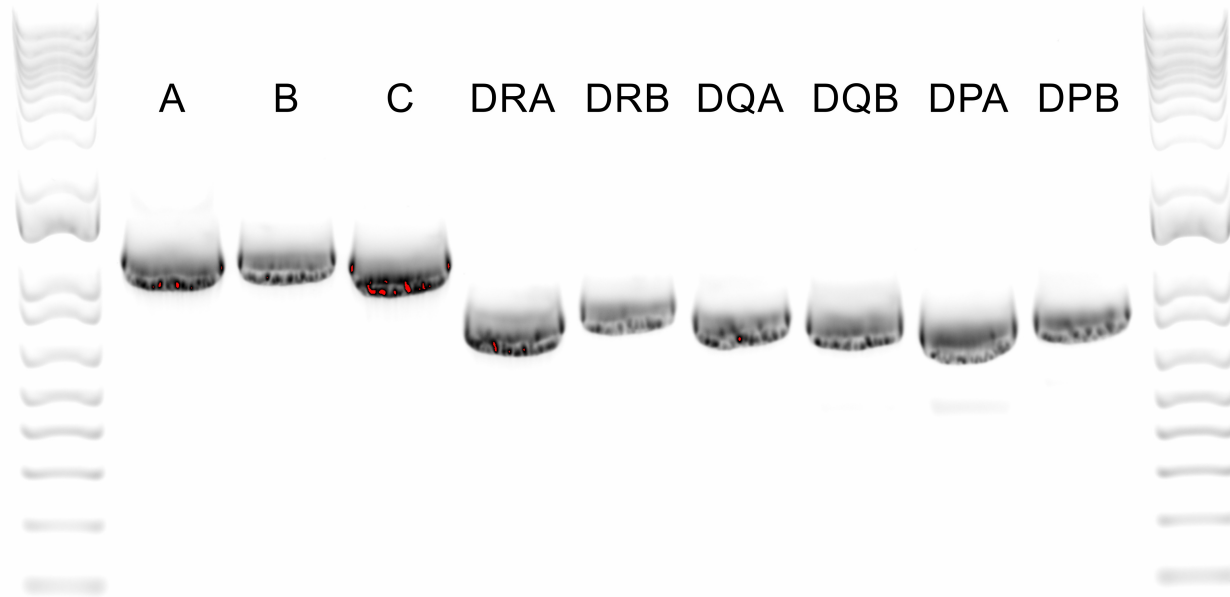

Supplement: S2 Appendix — (PDF) [file pone.0228112.s013.pdf]
